# Impact of zero‑output crisp range recentering on the performance of FPIDD^2^ controllers for multi-area LFC–AVR systems

**DOI:** 10.1038/s41598-026-61334-8

**Published:** 2026-07-17

**Authors:** Mohamed H. T. Omar, Ragi A. Hamdy, Hossam Kotb

**Affiliations:** Department of Electrical Engineering, Alexandria University, Alexandria, 21544 Egypt

**Keywords:** Fuzzy-based control strategy, FPIDD^2^ controller, Crisp-range configuration of fuzzy controller, Metaheuristic optimization techniques, Multi-area interconnected power network, LFC–AVR coordination, Energy science and technology, Engineering, Mathematics and computing

## Abstract

Modern interconnected power systems with high penetration of renewable energy sources (RES) and large‑scale use of electric vehicles (EVs) experience recurring frequency and voltage disturbances. Accordingly, the main objective in this work is to overcome this limitation in a power system under coordinated load frequency control (LFC) and automatic voltage regulation (AVR), which requires highly accurate control strategies. Fuzzy logic‑based controllers are among the most promising approaches for disturbance rejection, control precision, system stability, and robust performance. However, their performance is often limited because the selection of crisp ranges (i.e., the universes of discourse of the fuzzy variables) is usually set heuristically. In this paper, the crisp output range of a Fuzzy Proportional Integral Derivative Double Derivative (FPIDD^2^) controller, which is based on a previous study, is reconfigured while maintaining the original rule base and membership function structure unchanged. This reconfiguration is presented to change the controller’s response by recentering (shifting) the zero output membership function rightward, thereby improving control sensitivity and dynamic performance. The effectiveness of the proposed approach is validated via MATLAB simulation on a multi‑area interconnected power system under realistic operating conditions, including stochastic input fluctuations due to renewable energy source penetration and electric vehicle participation, as well as nonlinear constraints such as generation ramp‑rate limits and governor dead zones. Several metaheuristic optimizers, including Particle Swarm Optimization (PSO), Gorilla Troops Optimizer (GTO), and Marine Predators Algorithm (MPA), are used to further evaluate the robustness of the proposed method. The results demonstrate that optimized FLC configuration with modified crisp ranges significantly improves controller sensitivity, damping characteristics, and robustness. Consequently, the Integral of Time- Absolute Error (ITAE) is reduced by up to 69% compared to the original controller configuration.

## Introduction

### Background and research gap

Due to the increasing power demand, the proliferation of renewable energy sources (RESs), electric vehicle (EV) charging, and distributed generation, power systems are becoming increasingly complex^1–4^. This complexity leads to larger frequency and voltage variations in multi-area interconnected systems.

Therefore, it is crucial to have a robust and adaptive control strategy^5,6^. Intelligent control strategies, like fuzzy logic-based controllers, are very suitable to cope with the nonlinearities, uncertainties and fast dynamics that characterize modern power systems^7–9^. From this perspective, the hybrid Fuzzy Proportional Integral Derivative Double Derivative (FPIDD^2^) control scheme has proved to outperform classical PID and PIDD^2^ schemes. This is because FPIDD^2^ provides reduced settling time, improved damping and disturbance rejection in the context of coordinated load frequency control (LFC) and automatic voltage regulation (AVR), respectively^10–12^.

FPIDD^2^ controllers can be particularly effective in controlling frequency and voltage, providing robustness against large disturbances and high levels of renewable energy penetration. However, a key control design aspect which is generally neglected is the definition and tuning of the crisp input–output ranges in fuzzy controllers.

Most existing studies focus primarily on tuning controller gains, membership function shapes, or applying adaptive or optimization approaches, while the crisp ranges that define the universe of discourse of input and output variables and represent the numerical bounds within which fuzzy inference operates are typically selected heuristically or kept fixed through trial-and-error procedures^13–15^.

These quantified ranges govern the applicability of membership functions, the degree of rule firing, gain tuning, and overall controller sensitivity^16^. Overly narrow quantification of crisp input or output ranges may lead to poor dynamic performance, limited system robustness under large disturbances, or reduced accuracy in small-signal operating regions^17^. To date, no systematic and comprehensive investigation has examined the relationship between crisp range structures, such as narrow/wide or symmetric/asymmetric ranges and their effect on system dynamic stability or control quality^18^.

Moreover, crisp ranges have never been optimally configured using advanced metaheuristic algorithms in conjunction with other control design parameters, e.g., higher renewable power penetration, or incorporating system nonlinearities such as generation rate limits or governor deadbands^19,20^. To fill this gap, crisp configuration is taken into account in this paper as an important control design tuning parameter. The impact of crisp-range tuning on FPIDD^2^ controller performance is then investigated while maintaining the fuzzy rule base and membership function shape unchanged, which yields a general and reproducible methodology for enhancing multi-area power system robustness, scalability, and dynamic behavior.

### Literature review

Both LFC and AVR are considered the key mechanisms that are mandatory for ensuring frequency stability, voltage quality, and power balance in interconnected power systems. One of the Initial treatments of LFC and AVR coordination was presented by Sahu et al.^21^, who demonstrated the significance of these controls in counteracting the effect of frequency drops. An extensive discussion of coordinated LFC and AVR control for multi-area systems is provided by Alnefaie and El-Saadany^22^. Mohanty et al.^23^ and Shayeghi and Aliprantis^24^ established that system interconnection and its concomitant uncertainty in load demand leads to increased control requirements for both frequency and voltage stability.

The classical PID controllers have been traditionally used in LFC/AVR applications because of their simplicity and ease of implementation. Agarwal et al.^25^ pointed out that classical PID controllers suffer from degraded control performance during nonlinear operation and parameter variations. Abido^26^ also proved that PID-based LFC schemes are inadequate in attaining acceptable performance during RES integration with stochastic load variations. Srivastava and Gupta^27^ established that inter-area coupling effects and governor nonlinearities severely degrade fixed gain PID controllers’ robustness in present interconnected power networks.

Hence, these shortcomings led to the development of other control schemes. For example, fractional order control was proposed by Barros and Montoya^28^, who proved that fractional PID control has better configurability of system dynamics than integer-order PID control. Expanding this idea, Dash and Panda^29^ employed fractional order controllers to power system applications and observed better robustness and disturbance rejection. Recently, Singh et al.^30^ studied the adaptive intelligent control structure for LFC and AVR coordination and achieved better dynamic performance under varying operating conditions, and higher renewable penetration.

In recent years, fuzzy logic control has received much attention as a viable solution to modeling uncertainty and nonlinearity in power system control problems. Ray and Sahu^31^ established the mathematical basis of fuzzy logic. Al-Sulaiman^32^ demonstrated its usefulness in control applications. Panda^33^ demonstrated the ability of a fuzzy-based controller to compensate for nonlinearities in the power systems while accounting for parameter uncertainty.

Gupta and Srivastava^34^, Sahu^35^ have deployed fuzzy logic controllers for LFC problems, which demonstrated significant damping improvement and disturbance rejection. Dash^36^ further evaluated the performance of fuzzy and hybrid controllers in interconnected systems under high renewable penetration.

Chen et al.^37^ validated that fuzzy-based LFC–AVR schemes can improve system robustness to load and RES variations.

Khamari et al.^38^ established the effectiveness of fuzzy PID controllers over classical PID controllers by assessing transient response and robustness to load parameter variations. Othman and Ramdas^39^ proved the effectiveness of simple yet robust fuzzy PID controllers when employed in multi-area interconnected power systems. Satapathy et al.^40^ also confirmed the efficiency of fuzzy hybrid controllers in renewable-integrated power systems under stochastic load variations.

Wadi et al.^41^ demonstrated that these performance improvements of the fuzzy inference system are due to its ability to reason using linguistic rules, enhancing decision-making in power system control. Rasolomampionona et al.^42^ also reported that these improvements are due to the capability to map the input–output relationship nonlinearly.

Integrating fuzzy logic with more complex controller architectures has been investigated in recent studies of coordinated LFC–AVR applications. As a result, fuzzy logic is progressively integrated with the PIDD^2^ controllers to exploit the excellent damping properties of the derivative term of the PIDD^2^.

Jabari et al.^43^ suggested an advanced control technique that demonstrates better damping performance in LFC applications compared to the conventional PID controllers. Likewise, Dao et al.^44^ assessed how well a fuzzy PIDD^2^ controller deployed to enhance the dynamic performance of the power systems with strong RES penetration. In addition, fractional-order versions of the PIDD^2^ controller efficiently handled uncertainties as reported by Dai et al.^45^. Using fuzzy logic, Meseret and Bala Anand^46^ managed to control coordinated LFC–AVR power systems. Almost similar results were presented by AboRas et al.^47^, Esmaeili et al.^48^, and Alshehri et al.^49^, as the PIDD^2^ controllers succeeded to achieve better performance and faster responses compared to traditional controllers. Then, Mansour et al.^50^ combine fuzzy logic with PID controllers to achieve a faster response. These results are consistent with the findings of Shahzad et al.^51^. Similar results were reported by Gulzar et al.^52^. Abdelaziz et al.^53^ showed that hybrid configurations demonstrate robustness against GRC/GDB, which was further confirmed by Haldar et al.^54^, and later supported by Rahimi et al.^55^.

Even with the growing attention to fuzzy logic control, the systematic tuning of crisp input‑output ranges (i.e., the universes of discourse) have remained largely unexplored. As summarized in Table 1, existing studies focus on optimizing gains, rule bases, or membership function shapes, but the numerical bounds within which the fuzzy inference operates are usually set by heuristic rules of thumb or left unchanged.

**Table 1:** Literature comparison of fuzzy controller tuning methods highlighting the gap in crisp input–output range tuning.

Reference	Gains (K_p_, K_i_, K_d, scaling factors)	Rule base	Membership function (MF) shapes	Crisp (input/output) ranges
Sahu et al.^21^	✓	✗	✗	✗
Alnefaie and El-Saadany^22^	✓	✗	✗	✗
Mohanty et al.^23^	✓	✓	✗	✗
Ray and Sahu^31^	✓	✗	✗	✗
Panda^33^	✓	✗	✓	✗
Gupta and Srivastava^34^	✓	✗	✗	✗
Chen et al.^37^	✓	✓	✓	✗
Li et al.^38^	✓	✗	✗	✗
Kumar et al.^39^	✓	✗	✗	✗
Jabari et al.^43^	✓	✗	✗	✗
Sharma et al.^44^	✓	✗	✗	✗
Singh et al.^46^	✓	✓	✓	✗
This work	✓ (via optimizers)	✗ (fixed)	✗ (fixed)	✓ (zero output range recentered)

To address this specific gap, the present study investigates the effect of recentering the zero output crisp range as a controlled design variable for fuzzy PIDD^2^ controllers in coordinated LFC‑AVR applications. Unlike previous works, this study deliberately keeps the rule base and all membership function shapes fixed, while only one crisp range (the zero output MF) is adjusted. Accordingly, this work could be considered the first systematic investigation of crisp‑range recentering in FPIDD^2^ controllers for multi‑area LFC‑AVR systems.

Many optimization techniques have been deployed to optimize the performance of LFC–AVR controllers. For instance, PSO^56^ was proposed for global optimization problems, especially in power system control applications. Kumar et al.^57^ demonstrated the effectiveness of PSO for LFC controller tuning, while Shahzad et al.^58^ worked on how to improve the convergence characteristics of the PSO algorithm. Afterwards, more recent algorithms were deployed in modern power system optimization studies. As an example, Kumar et al.^57^ proposed a GTO-based optimization for LFC. In addition, Shahzad et al.^58^ proposed an MPA-based cascaded controller tuning approach for LFC control in a hybrid power system. Khamari^59^ checked modeling and performance prediction methods for PV generation. Bhatti et al.^60^ investigated the impact of governor dead band on power systems. Hou and Xiong^61^ developed modeling of generation rate constraints in multi-area power systems. Morsali et al.^62^ studied control of multi-area power systems with hybrid generation. Parmar et al.^63^ proposed nonlinear modeling and controller design techniques for power systems integrated with RES. Nahas et al.^64^ implemented an automatic voltage regulation technique for power systems incorporating RES. Khamari et al.^65^ developed dynamic PV generation models considering weather-dependent effects. Elkasem et al.^66^ modeled wind power generation using a stochastic white noise–based simulation technique. Saikia and Das^67^ analyzed load frequency control of a two-area multi-source power system with electric vehicles. Aly et al.^68^ proposed an optimized fractional-order load frequency control strategy for interconnected microgrids with high renewable and EV penetration.

Several recent studies have demonstrated the value of rigorous, statistically validated metaheuristic-based tuning for advanced controllers in nonlinear power and industrial systems. Jabari et al.^43^ introduced a bio dynamic grasshopper optimization algorithm (BDGOA) to tune a multi stage TDn (1 + PI) controller for load frequency control. Similarly, Jabari et al.^69^ proposed a TDn (1 + PIDn) controller optimized by the diligent crow search algorithm (DCSA) for nonlinear steam condenser pressure regulation. Izci et al.^70^ combined a 2DOF PIDA structure with the starfish optimization algorithm (SFOA) for temperature regulation in a continuous stirred tank heater (CSTH). These works exemplify the importance of reporting mean, standard deviation, and significance testing when using stochastic optimizers, a practice that is adopted in the present study (see “Statistical framework for performance assessment” section) to ensure the credibility of reported improvements.

### Paper organization and contribution

The next sections of this paper are structured as follows:

- “Investigated system” section presents a simple presentation of the multi area power system model with RES and EV integration.
- “Proposed controllers” section shows FPIDD^2^ controller configuration and the fine-tuning methodology of its crisp range.
- “Case studies” section illustrates the simulation results and comparative performance evaluation.
- “Statistical framework for performance assessment” section demonstrates the statistical framework for performance assessment.
- “Conclusion” section provides the paper’s final conclusion and suggests areas for future research.

This study is a direct extension of our previous work^71^. To ensure a fair and interpretable comparison, the following elements from^71^ were kept without modification:

- The two-area interconnected power system model, including all generation units (thermal, gas, hydro), renewable energy sources (PV, wind), EV aggregators, AVR dynamics, and nonlinear constraints (GRC, GDB).
- The FPIDD^2^–PIDD^2^ control architecture (FPIDD^2^ for LFC, PIDD^2^ for AVR).
- The fuzzy rule base and all membership function shapes for the Initial FLC (except the crisp range of the zero output MF, which is modified).
- The three metaheuristic optimizers (PSO, GTO, MPA) and their parameter bounds.
- The optimization settings (population size = 20, maximum iterations = 30).

The new contributions introduced in this paper are:

- Implementation of a novel approach to fine-tune the controller by shifting only the Zero output membership function (zero‑output crisp range recentering) to improve performance without modifying the existing FLC rule base or other membership functions.
- Examining the role of the ITAE fitness function in shaping both transient and steady-state system responses, while validating its effectiveness in minimizing long-term error fluctuations and improving overall dynamic performance.
- Integration with metaheuristic optimization using PSO, MPA, and GTO to evaluate and enhance crisp range tuning (zero-output membership function recentering) and to assess their effectiveness in improving the stability and control performance of coordinated LFC–AVR systems.
- Validating operational conditions by highlighting the resilience of the proposed controller with regard to RES intermittency and EVs integration, and its robustness under nonlinear system constraints (GRC, and GDB).

In this work, ITAE can be significantly reduced by simply shifting the zero-output crisp range, while keeping all other parameters of FLC. Moreover, the methodology is general and can be applied to any fuzzy logic controller, regardless of the specific plant or control objective. Therefore, this technique provides significant added value beyond^71^ while maintaining a clean, controlled experimental setup.

This work is among the first studies that systematically investigate the effect of zero‑output crisp range positioning as an independent design variable in FPIDD^2^ controllers deployed in coordinated LFC–AVR systems. Accordingly, this study reveals a tuning dimension that has been systematically ignored in the fuzzy control literature, as evidenced by the comparative summary in Table 1.

All results presented in this work are obtained from high-fidelity simulations. Therefore, experimental, hardware, and real-time validation are beyond the current scope and are identified as necessary future steps. However, the simulation methodology and findings provide a strong foundation for such future validation.

In addition, the scope of this study is limited to demonstrating the value of crisp-range tuning within a fixed FPIDD^2^ architecture. Comparisons with other controller families (e.g., adaptive, MPC, and AI-based approaches) are not included. However, the proposed methodology and findings provide a basis for future comparative investigations.

## Investigated system

### System under study

**Fig. 1:**
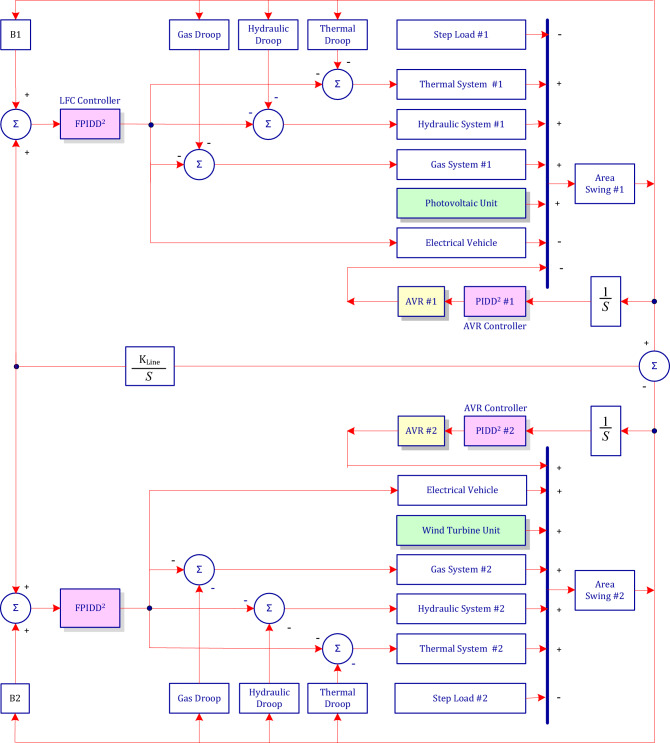
Simplified block diagram of the investigated two-area interconnected power system with aggregated conventional generation, RES units, EV aggregators, and coordinated LFC–AVR control loop.

In this paper, a simplified block diagram of the overall system configuration is presented in Fig. 1, while full technical details are provided in^71^. The investigated two-area interconnected power system considered in this study follows the same modeling framework presented in our previous work^71^. Readers familiar with^71^ will recognize that the plant model remains unchanged; only the controller’s crisp range is modified.

## Proposed controllers

### Controllers’ structures

The overall control architecture adopted in this study follows the framework reported in our previous work^71^, where FPIDD^2^ controllers are employed in the LFC loops, while PIDD^2^ controllers are utilized in the AVR loops. The FPIDD^2^ controller structure implemented herein is based on the same design methodology presented in^71^. Accordingly, it receives two input signals, namely the present error (E) and the change in error (CE), and employs five linguistic variables: Large Negative (LN), Small Negative (SN), Zero (Z), Small Positive (SP), and Large Positive (LP). Although this linguistic set has demonstrated acceptable performance, a higher number of linguistic variables can be used to achieve improved control resolution and accuracy.

The detailed controller structure, block diagrams, fuzzy rule base, and mathematical expression of the FPIDD^2^ control law are not repeated here to avoid redundancy. However, interested readers are referred to^71^ for the complete theoretical development and implementation.

The fuzzy logic controller (FLC) within the FPIDD^2^ framework is implemented in two configurations, referred to as Initial FLC and Enhanced FLC. These configurations differ only in the definition of the crisp output ranges. In the Initial FLC, the ranges are selected based on standard rules of thumb, as suggested in^71^. While these settings provide a reasonable starting point, they may limit the effective utilization of the membership functions and reduce inference resolution. The Enhanced FLC modifies only the crisp output ranges, while all other controller parameters remain unchanged.

By isolating the crisp range as the sole design variable, the impact of this modification can be clearly evaluated through comparison between the Initial and Enhanced FLC configurations.

### Enhanced FLC

The Enhanced FLC deployed in the FPIDD^2^ platform is obtained by modifying only the crisp range of the zero (Z) output membership function (a specific recentering strategy), while all other controller components, including membership function shapes, rule base, inference mechanism, and defuzzification strategy, are maintained identical to those of the Initial FLC.

**Fig. 2:**
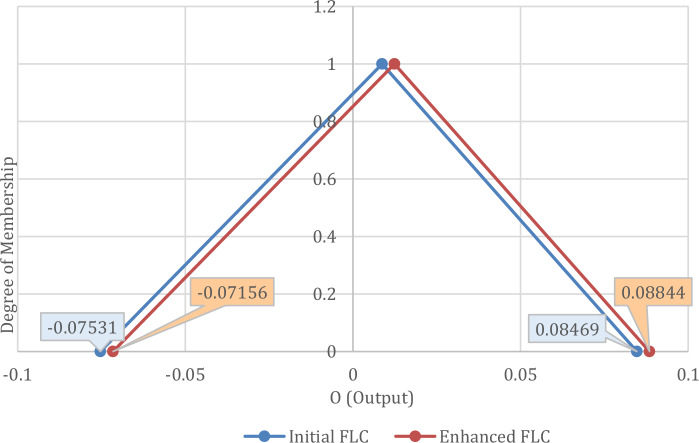
Zero (Z) output membership function for Initial and Enhanced FLCs.

Specifically, the crisp range of the Z output membership function is shifted from [− 0.07531, 0.00871, 0.08469] to be [− 0.07156, 0.01241, 0.08844], which corresponds to an approximately uniform rightward shift of + 0.00375, while preserving the crisp range span as indicated in^71^. A quantitative comparison between the crisp ranges of the zero (Z) output membership function for the Initial and Enhanced FLCs is reported in Fig. 2 and Table 2.

**Table 2:** Comparison between the crisp range of the zero (Z) output membership function for Initial and Enhanced FLCs.

Fuzzy variable	MF used	Crisp range for (Z) of	
		Initial FLC	Enhanced FLC
Output (O)	Triangular MF	[− 0.07531, 0.00871, 0.08469] (Span: 0.16000)	[− 0.07156, 0.01241, 0.08844] (Span: 0.16000)

Figure 2 as well as Table 2, show the configurations of both the Initial FLC and Enhanced FLC. Although the span of both controllers is kept the same with a value of (0.16000), the crisp range of the Zero (Z) membership function differs.

The proposed configuration applied to the Enhanced FLC intentionally shifts the core of the Zero membership function within the positive region of the output. The inclusion of the compensatory offset in the control law can help minimize some of the inherent biases and asymmetries in the system’s dynamic response that are caused by nonlinear effects like offsets in the sensors, dead zones in the actuators, and static friction. As a result, the Enhanced FLC can effectively interpret the linguistic concept of “Zero” as a small positive corrective action and so provides a lower steady-state error, reduced oscillations, and better setpoint tracking.

From the viewpoint of control system analysis, relocating the zero-output membership function acts similarly to a rule-dependent adaptive gain adjustment^72^. This approach rescales the local input–output relationship of the controller, and so increases the proportional-like gain and alters the describing function of the fuzzy controller near the equilibrium point when close to zero error, while nonlinear effects are kept in larger deviations. These changes improve responsiveness to small disturbances without compromising stability during large transients, resulting in sensitivity improvements and greater restoring action close to equilibrium with neither extra phase lag nor reduction in stability margins^73,74^.

In this case, the direction (sign) of the crisp range shift is very crucial. As an example, a rightward shift will enhance the corrective action for small positive errors, and speed convergence. Whereas, a leftward shift would have the opposite effect, and might degrade transient response^75,76^. Accordingly, the Zero output crisp range could be considered one of the most critical tuning parameters of FLC performance.

To further substantiate this observation, a parametric sensitivity analysis was performed to rigorously validate the causal role of the shift magnitude and direction. The zero-output crisp range was shifted by varying amounts shown in Table 3, while keeping all other controller parameters fixed. The same test conditions as Case Study 1 (MPA optimizer) were applied.

**Table 3:** Tested zero-output crisp range shifts of FLC applied to assess magnitude and direction effects on performance.

No	Shift amount	Description	Resultant ITAE
1	− 0.0075	Leftward (opposite direction), double magnitude	67.9
2	− 0.00375	Leftward (opposite direction), same magnitude	223.5
3	0	No shift (the original Initial FLC)	51.6
4	+ 0.00375	The proposed shift	11.1
5	+ 0.0075	Rightward, double magnitude	19.26
6	+ 0.01125	Rightward, triple magnitude	17.9

As illustrated in Table 3, the shift magnitude =  + 0.00375 was not chosen arbitrarily, as a systematic parametric sweep was performed to determine the optimal rightward shift of the zero-output crisp range. In this sweep, the crisp range of the zero-membership function was modified by adding a shift to all three points of the triangular MF. For each shift, the controller gains were kept fixed at the values optimized for the original FLC, while the crisp range was varied.

**Fig. 3:**
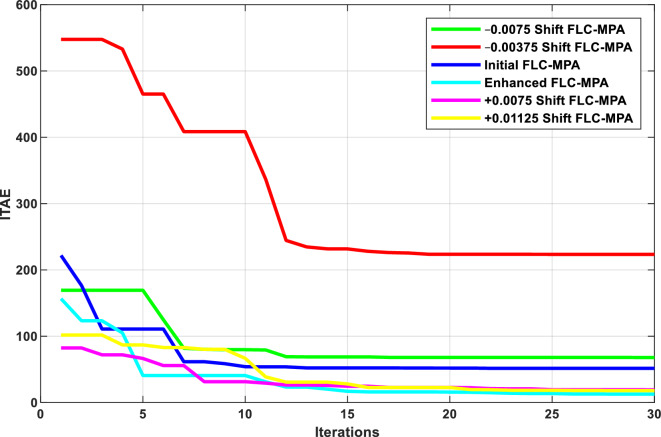
ITAE values of tested zero-output crisp range shifts of the FLC.

Figure 3 illustrates the ITAE variation as a function of the zero-output crisp range shift. The results indicate that negative (leftward) shifts consistently degrade performance by increasing ITAE. For positive shifts, a rapid reduction in ITAE is observed at shift =  + 0.0075, after which performance deteriorates, with a noticeable increase at shift (+ 0.01125). Meanwhile, the selected value (shift =  + 0.00375) lies within the region of steep improvement and is close to the optimal operating point for the considered system. This non-monotonic behavior confirms that small positive shifts enhance performance, whereas excessive shifting leads to overcompensation and the emergence of a steady-state error of opposite sign.

From a control-theoretic perspective, the rightward shift of the zero-output membership function (+ 0.00375) modifies the local control gain of the fuzzy inference system in the vicinity of the equilibrium point. Let the fuzzy control output be expressed as control output (error, error derivative). For small error conditions (error ≈ 0, error derivative ≈ 0), a symmetric zero membership function yields control output ≈ 0. However, introducing a rightward shift induces a small positive control action even at zero error, effectively introducing a controlled bias. This bias compensates for inherent system asymmetries such as GDB, static friction in turbine valves, and steady offsets arising from renewable energy integration. Consequently, the controller exhibits improved sensitivity to small-magnitude disturbances, effectively increasing the local proportional-like action around the setpoint.

Nevertheless, excessive shifting (e.g., ≥  + 0.01125) results in an overly dominant positive bias at equilibrium, which introduces sustained offset and degrades steady-state accuracy. This explains the non-monotonic ITAE trend observed in Fig. 3, where an optimal shift magnitude exists. Accordingly, for the investigated multi-area LFC–AVR system, shift =  + 0.00375 provides the best compromise between rapid error attenuation and steady-state accuracy.

Based on the observed behavior in Fig. 3, the same tuning strategy can be generalized as follows:

(i) Initialize a baseline fuzzy controller with gains optimized by any suitable method;
(ii) Perform a one-dimensional parametric sweep of the zero-output crisp range shift over both negative and positive values using small increments; and
(iii) Select the shift value that minimizes the ITAE or an equivalent performance index. Since only a single membership function is adjusted, this procedure introduces negligible computational overhead compared to full-scale gain optimization techniques.

To conclude, the recommended rightward shift of the crisp interval of the Zero output membership function could form a scientific base for enhancing FLC performance within the FPIDD^2^ framework^72–74^.

**Fig. 4:**
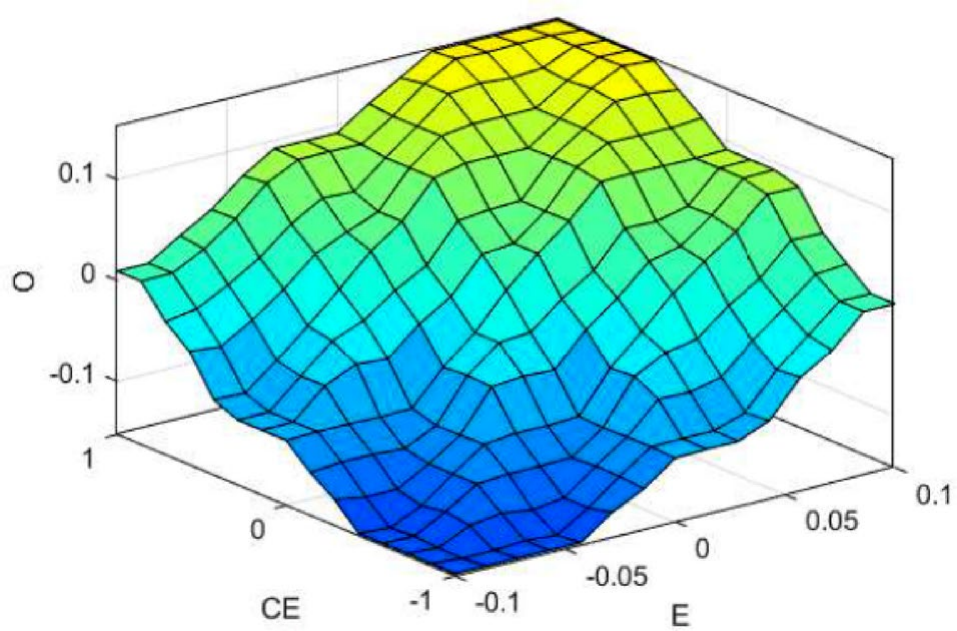
Rule surface viewer of the enhanced FLC.

**Fig. 5:**
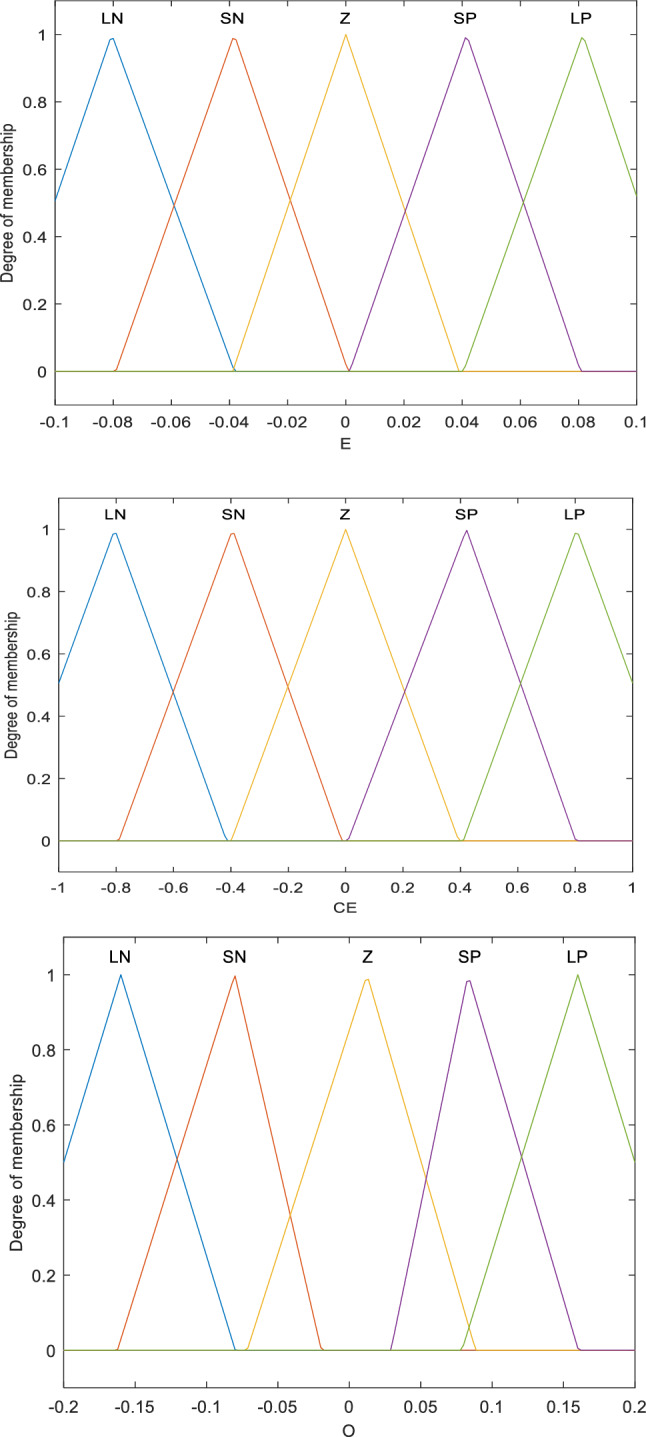
Membership functions for the enhanced FLC.

The three-dimensional rule surface of the Enhanced FLC is presented in Fig. 4, whereas the input and output membership functions of the Enhanced FLC, are presented in Fig. 5.

### Optimization methods

The optimization algorithms that are used in this research, namely Particle Swarm Optimization (PSO), Gorilla Troops Optimizer (GTO), and Marine Predators Algorithm (MPA) are specifically chosen to be the same as in our published research^71^, where they are used as benchmarks to assess the performance of other algorithms. This allows a fair comparison between the performance of the Initial FLC described in^71^ and the proposed Enhanced FLC developed herein.

Moreover, the comparative study has also been performed by using the same set of optimizers, parameter bounds, population size, number of iterations and convergence criterion. Consequently, the obtained results provide a fair basis for evaluating the effectiveness and robustness of the proposed FLC, while also enabling reproducibility and direct comparison with the earlier results presented in^71^.

### Comparative evaluation methodology

Table 4 presents a summarized simulation methodology adopted to provide a comparative evaluation of the two FLC configurations (Initial and Enhanced). Both arrangements are included inside the hybrid FPIDD^2^–PIDD^2^ control architecture and deployed in a two-area interconnected power system. The main goal of this work is to evaluate the dynamic behavior of the proposed Enhanced FLC arrangement against that of the Initial FLC configuration already presented in our past research^71^. This is done by preserving the identical controller structure while applying only the crisp-range modification to identify the exact contribution of crisp-range adaptation to overall control performance.

**Table 4:** Optimization trials and controller configurations for multi-area power system.

Trial	Attempt	Experiment	Optimizer	LFC controller		AVR CONTROLLER	
				Area 1	Area 2	Area 1	Area 2
First	1	1	PSO	Initial FPIDD^2^	Initial FPIDD^2^	PIDD^2^	PIDD^2^
	2	2	GTO	Initial FPIDD^2^	Initial FPIDD^2^	PIDD^2^	PIDD^2^
	3	3	MPA	Initial FPIDD^2^	Initial FPIDD^2^	PIDD^2^	PIDD^2^
Second	1	4	PSO	Enhanced FPIDD^2^	Enhanced FPIDD^2^	PIDD^2^	PIDD^2^
	2	5	GTO	Enhanced FPIDD^2^	Enhanced FPIDD^2^	PIDD^2^	PIDD^2^
	3	6	MPA	Enhanced FPIDD^2^	Enhanced FPIDD^2^	PIDD^2^	PIDD^2^

The Enhanced FPIDD^2^ is based on the Enhanced FLC architecture, while the Initial FPIDD^2^ includes the Initial FLC structure. For simplicity in the next sections, the results of FPIDD^2^ + PIDD^2^ controller scheme based on the Initial FLC are termed the “Initial FLC”, whereas the results of the same configuration incorporating the Enhanced FLC are referred to as the “Enhanced FLC”.

The assessment is divided into two trials, each one of them includes four operating scenarios. In the first trial, the FPIDD^2^–PIDD^2^ control scheme uses the Initial FLC, following the same architecture described in^71^. Three separate optimization runs with each one based on a metaheuristic algorithm including PSO, GTO, and MPA, respectively. In the second trial, the same FPIDD^2^–PIDD^2^ architecture is employed, but the Initial FLC is replaced by the Enhanced FLC.

To ensure comparability, methodological fairness, and robustness of the performance trends, an identical tuning protocol is applied across all six optimization experiments (two trials × three optimizers).

The present study focuses exclusively on comparing the proposed Enhanced FLC, which incorporates zero-output crisp range recentering, against the Initial FLC baseline from^71^. This controlled setup is adopted to isolate the effect of crisp range tuning and avoid confounding factors.

Accordingly, no direct comparison is made with other advanced control strategies such as adaptive control, model predictive control, sliding mode control, neural network-based controllers, reinforcement learning approaches, or type-2 fuzzy logic systems. While such comparisons would provide broader benchmarking context, they are beyond the scope of this study. The primary contribution here is to demonstrate that a simple and often overlooked tuning parameter, namely the zero-output crisp range, can yield substantial performance improvements within an existing controller architecture. Comprehensive benchmarking against other controller classes is therefore deferred to future work.

### Fitness function and parameter constraints

Performance indices play a significant role in the area of control system design, as they provide a quantitative measure (score) of how well the system is performing in response to a perturbation and achieving the desired performance. The use of performance indices is of significant importance in assessing the performance of a system in terms of its transient as well as steady-state response, which can guide controller design and tuning toward optimal system operation. Various types of performance indices could be employed to assess the performance of a system. Among these, it is found that the use of Integral of Time Absolute Error (ITAE) is mostly preferred, as it is capable of balancing response rate, smoothness, as well as damping. This is achieved by imposing a heavier weight on those errors that tend to be sustained over time compared to Initial errors, thereby effectively dampening oscillations, reducing steady-state error, and so enhancing overall stability. Because of such advantages, ITAE has become a standard objective function in many modern control loops and so it is extensively applied in studies on LFC and AVR systems^75–77^. In this study, each controller’s performance is assessed using ITAE, following the same methodology as presented in the previous study^71^ to ensure consistency in evaluation and to enable reliable comparison across different control strategies. The mathematical expression of Integral of Time Absolute Error (ITAE) is presented in Eq. (1), while Table 5 illustrates the parameter constraints and bounds used for tuning both the LFC and AVR loops.

1 $$Integral\;time\;absolute\;error\left( {ITAE} \right) = \mathop \smallint \limits_{0}^{{T_{simulation} }} \left( {\left| {\Delta V_{1} } \right| + \left| {\Delta V_{2} } \right| + \left| {\Delta P_{tie} } \right| + \left| {\Delta F_{1} } \right| + \left| {\Delta F_{2} } \right|} \right)*t dt$$

**Table 5:** Parameter constraints and bounds for LFC and AVR tuning.

Parameter	Parameter constraints	Load frequency control bounds		Automatic voltage regulation bounds	
		Lower bound	Upper bound	Lower bound	Upper bound
${\mathrm{K}}_{{\mathrm{P}}}$	${\mathrm{K}}_{{{\mathrm{P}}\;{\mathrm{Lower}}}} \le {\mathrm{K}}_{{\mathrm{P}}} \le {\mathrm{K}}_{{{\mathrm{P}}\;{\mathrm{Upper}}}}$	0.10	100.0	0.10	2.00
${\mathrm{K}}_{{\mathrm{I}}}$	${\mathrm{K}}_{{{\mathrm{I}}\;{\mathrm{Lower}}}} \le {\mathrm{K}}_{{\mathrm{I}}} \le {\mathrm{K}}_{{{\mathrm{I}}\;{\mathrm{Upper}}}}$	0.10	2.00	0.10	2.00
${\mathrm{K}}_{{{\mathrm{D}}1}}$	${\mathrm{K}}_{{{\mathrm{D}}1\;{\mathrm{Lower}}}} \le {\mathrm{K}}_{{{\mathrm{D}}1}} \le {\mathrm{K}}_{{{\mathrm{D}}1\;{\mathrm{Upper}}}}$	0.10	2.00	0.10	2.00
${\mathrm{K}}_{{{\mathrm{D}}2}}$	${\mathrm{K}}_{{{\mathrm{D}}2\;{\mathrm{Lower}}}} \le {\mathrm{K}}_{{{\mathrm{D}}2}} \le {\mathrm{K}}_{{{\mathrm{D}}2\;{\mathrm{Upper}}}}$	0.10	10.00	0.10	2.00
${\mathrm{N}}_{1}$	${\mathrm{N}}_{{1\;{\mathrm{Lower}}}} \le {\mathrm{N}}_{1} \le {\mathrm{N}}_{{1\;{\mathrm{Upper}}}}$	0.10	2.00	0.10	2.00
${\mathrm{N}}_{2}$	${\mathrm{N}}_{{2\;{\mathrm{Lower}}}} \le {\mathrm{N}}_{2} \le {\mathrm{N}}_{{2{\mathrm{Upper}}}}$	0.10	100.0	0.10	100.0
${\mathrm{K}}_{{\mathrm{E}}}$	${\mathrm{K}}_{{{\mathrm{E}}\;{\mathrm{Lower}}}} \le {\mathrm{K}}_{{\mathrm{E}}} \le {\mathrm{K}}_{{{\mathrm{E}}\;{\mathrm{Upper}}}}$	0.01	2.00	–	–
${\mathrm{K}}_{{{\mathrm{CE}}}}$	${\mathrm{K}}_{{{\mathrm{CE}}\;{\mathrm{Lower}}}} \le {\mathrm{K}}_{{{\mathrm{CE}}}} \le {\mathrm{K}}_{{{\mathrm{CE}}\;{\mathrm{Upper}}}}$	0.01	0.10	–	–

Table 3 summarizes the parameter constraints and search bounds used for tuning the LFC and AVR controllers. The symbol ‘–’ indicates that the corresponding parameters are not included in the AVR controller formulation. These bounds define the permissible search space for all optimization algorithms.

All optimization algorithms were initialized using random values drawn from a uniform distribution within the prescribed bounds of each controller parameter.

To ensure consistency and fair comparison across all optimization techniques, the number of search agents was fixed at 20, while the maximum number of iterations was set to 30. Accordingly, each optimization run was terminated at the 30th iteration, following the same configuration adopted in the previous study^71^.

These settings represent a practical trade-off between computational effort and solution quality. A sensitivity analysis (“Statistical framework for performance assessment” section) confirms that the main conclusions, namely the superiority of the Enhanced FLC and the relative ranking of optimization algorithms, remain valid across a reasonable range of population sizes and iteration limits, provided that the computational budget is not excessively reduced (for example, population size less than 20 or iteration limit less than 30). However, higher computational budgets can further improve the ITAE performance, which is identified as a potential direction for future refinement.

## Case studies

### Case study 1: impact of step load disturbances

#### System under study

This case study depends on the same two-area interconnected system presented in Fig. 1, but without renewable energy source (RES) integrated. Taking into account that a load perturbation (SLP) is set at 2.5% and applied in the first area at time at 50 s and in the second area at 150 s.

#### Simulation results

**Fig. 6:**
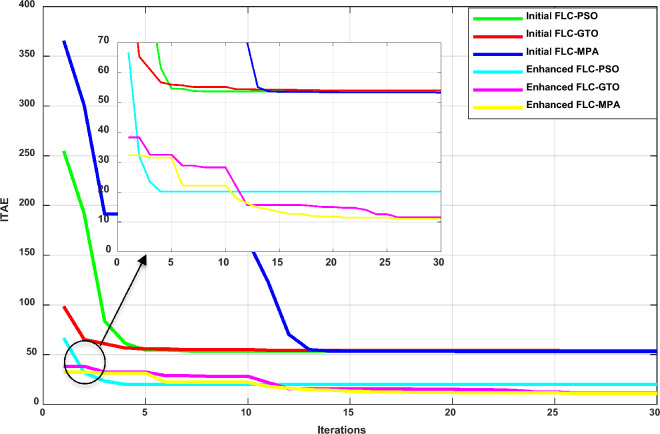
Convergence profile of the ITAE obtained during the optimization of controller parameters in the 1st case study. The curves correspond to: Initial FLC–PSO (Green), Initial FLC–GTO (Red), Initial FLC–MPA (Blue), Enhanced FLC–PSO (Cyan), Enhanced FLC–GTO (Magenta), and Enhanced FLC–MPA (Yellow).

**Fig. 7:**
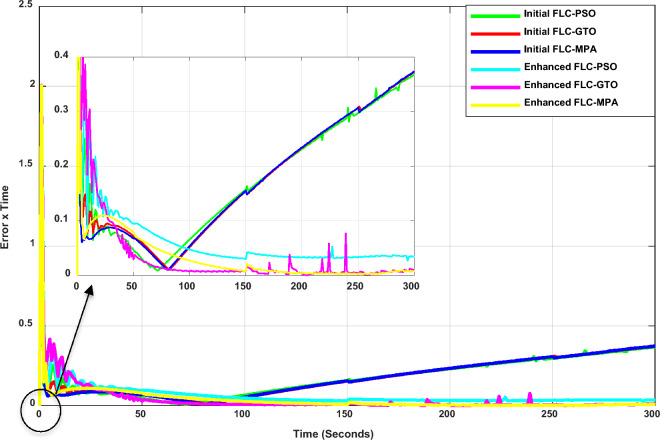
The ITAE Integrand (Error × Time) during controller performance evaluation in Case Study No.1. The curves correspond to: Initial FLC–PSO (Green), Initial FLC–GTO (Red), Initial FLC–MPA (Blue), Enhanced FLC–PSO (Cyan), Enhanced FLC–GTO (Magenta), and Enhanced FLC–MPA (Yellow).

**Fig. 8:**
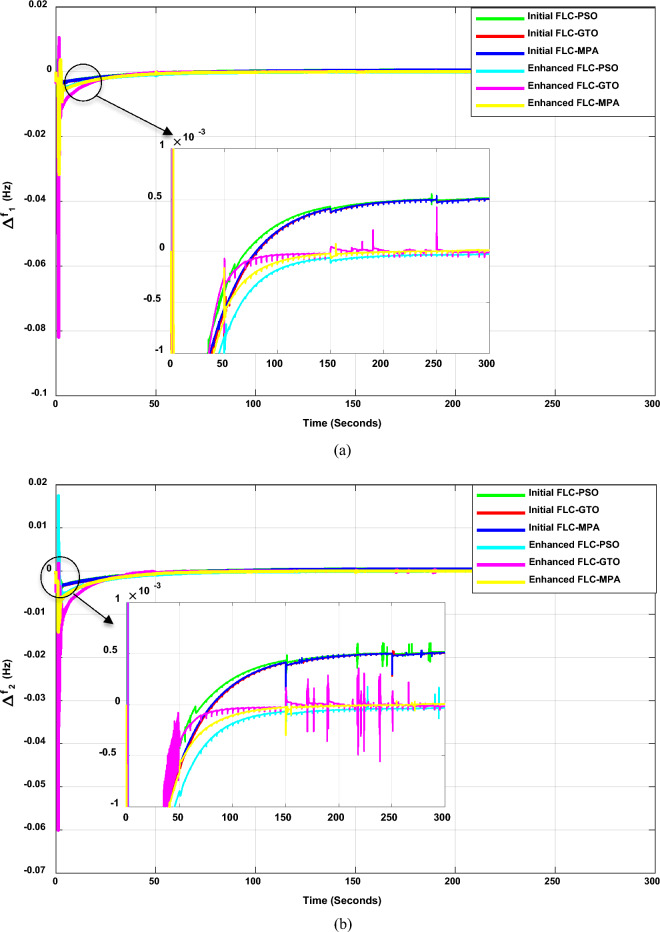

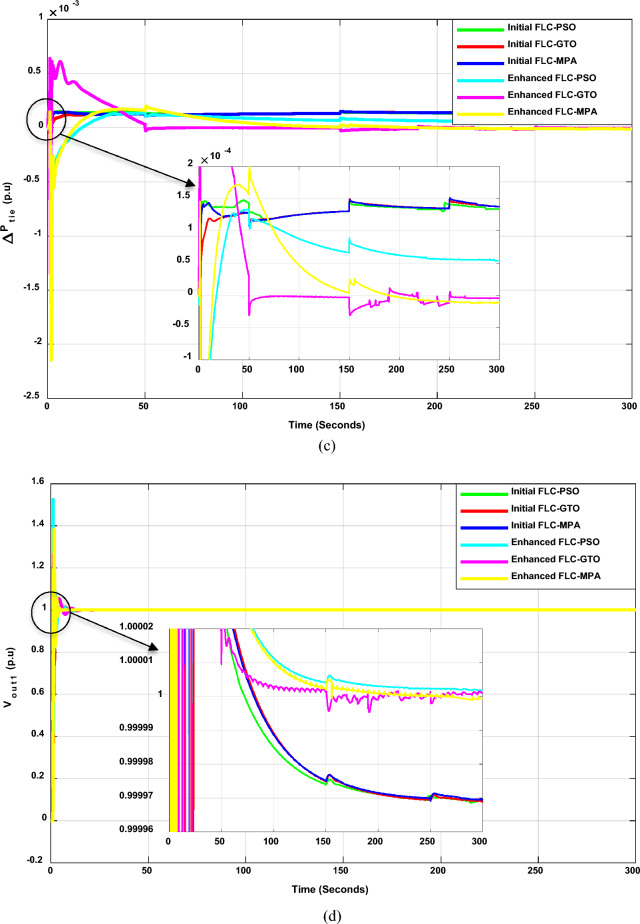

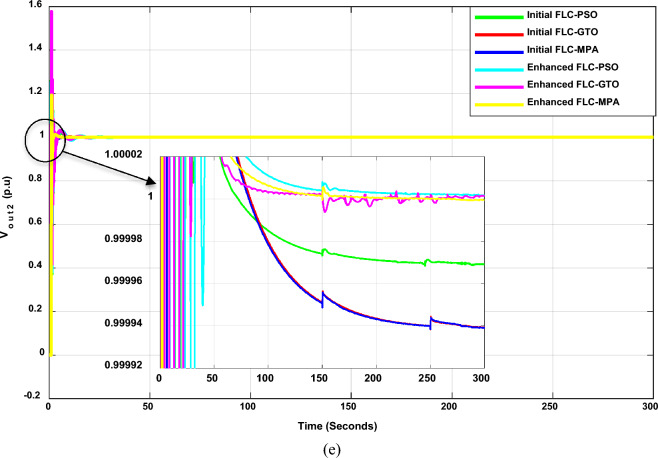
Time-domain responses of the two-area interconnected system under the 1st case study: (**a**) ∆f_1_, (**b**) ∆f_2_, (**c**) ΔP_tie_, (**d**) V_out1_, (**e**) V_out2_. The curves correspond to: Initial FLC–PSO (Green), Initial FLC–GTO (Red), Initial FLC–MPA (Blue), Enhanced FLC–PSO (Cyan), Enhanced FLC–GTO (Magenta), and Enhanced FLC–MPA (Yellow).

Figures 6 and 7 illustrate the optimization convergence profiles. In this context, Fig. 6 indicates the optimization criterion for ITAE, while Fig. 7 indicates the optimization criterion for the product of error and time. Moreover, Fig. 8 indicates the transient and steady-state response of the power system dynamics.

As shown in Fig. 6, the Enhanced FLC has better performance than the Initial FLC controllers. On one hand, the Initial FLC controllers converge with low convergence rates and have large ITAE values of about 50 resulting in large steady-state errors. On the other hand, the Enhanced FLC has shown a significant improvement in the convergence rate. In particular, the Enhanced FLC optimized using the MPA and GTO has a rapid convergence rate with an ITAE value of about 10 and negligible steady-state error. Moreover, the PSO-optimized Enhanced FLC has shown significant improvement in the performance of the Initial FLC controllers with an ITAE value of about 20.

In addition, as shown in Fig. 7, the Initial FLC controllers show slow rates of convergence and large steady-state errors. As a result, the integral errors build up over time, showing a poorer ability in disturbance rejection and dynamic control. On the other hand, it is seen that the Enhanced FLC controllers show a significantly improved response in both transient and steady states under all optimization techniques.

As shown in Fig. 8, a considerable difference is found between the performance of frequency deviations (Δf_1_ and Δf_2_) of the two FLC controllers under test. In this case, the Initial FLC controllers showed slow recovery and poor damping characteristics, while the Enhanced FLC controllers showed a better stability with almost zero steady-state error.

Regarding tie-line power deviations (ΔP_tie_), the Initial FLC controller provides sluggish convergence, which reflects weak disturbance rejection and poor inter-area synchronization. On the other hand, the Enhanced FLC controllers, achieve better stability, taking into account that the Enhanced FLC tuned using MPA provides the most effective damping with negligible overshoot, and lowest steady-state deviation.

Regarding the voltage responses in both areas (V_out1_ and V_out2_), the Initial FLC controllers exhibit significant voltage deviations. In comparison, the Enhanced FLC controllers display much better performance, with faster recovery, and minimal deviation. Again, in terms of delivering the most robust response, the Enhanced FLC-MPA configuration outperforms the rest (PSO, and GTO).

In conclusion, the Enhanced FLC scheme provided improved damping and robustness. Regarding the optimization methods, MPA achieved the overall best performance, while GTO is ranked second with almost equal performance, and PSO comes next.

The optimized parameter values identified for the 1st case study are summarized in Tables 6 and 7, while the corresponding time-domain dynamic performance indicators of the power system are reported in Table 8.

**Table 6:** Optimized tuning parameters of the LFC controllers for both interconnected areas.

Optimizer	Area	Initial FLC								Enhanced FLC							
		K_P_	K_I_	K_D1_	K_D2_	N_1_	N_2_	K_E_	K_CE_	K_P_	K_I_	K_D1_	K_D2_	N_1_	N_2_	K_E_	K_CE_
PSO	Area 1	100.0	2.00	2.00	8.50	2.00	100.0	2.00	0.10	51.20	1.70	2.00	8.98	1.60	100.0	1.92	0.10
	Area 2	100.0	2.00	2.00	10.00	2.00	100.0	2.00	0.10	77.27	1.90	2.00	3.85	1.92	97.10	2.00	0.10
GTO	Area 1	100.0	2.00	1.67	10.00	1.93	91.13	2.00	0.08	31.88	1.78	0.85	4.99	1.32	47.93	1.64	0.06
	Area 2	100.0	2.00	2.00	10.00	0.71	100.0	2.00	0.02	39.69	1.90	1.54	6.61	1.71	37.21	1.12	0.09
MPA	Area 1	100.0	2.00	1.93	2.25	2.00	99.97	2.00	0.09	37.51	1.77	1.44	7.76	0.29	92.10	1.99	0.04
	Area 2	100.0	2.00	1.99	10.00	1.97	92.78	2.00	0.04	67.52	1.73	2.00	9.89	1.74	86.32	1.95	0.01

**Table 7:** Optimized tuning parameters of the AVR controllers for both interconnected areas.

Optimizer	Area	Initial FLC								Enhanced FLC							
		K_P_	K_I_	K_D1_	K_D2_	N_1_	N_2_	K_E_	K_CE_	K_P_	K_I_	K_D1_	K_D2_	N_1_	N_2_	K_E_	K_CE_
PSO	Area 1	2.00	2.00	2.00	1.93	2.00	100.0	–	–	2.00	2.00	1.79	2.00	2.00	72.05	–	–
	Area 2	1.83	2.00	2.00	2.00	2.00	100.0	–	–	1.25	2.00	2.00	2.00	2.00	100.0	–	–
GTO	Area 1	1.99	1.97	2.00	2.00	1.99	100.0	–	–	0.95	1.70	1.48	1.00	0.92	33.52	–	–
	Area 2	2.00	2.00	1.40	1.08	2.00	100.0	–	–	1.33	1.44	1.41	1.88	1.55	42.65	–	–
MPA	Area 1	1.99	2.00	2.00	0.70	1.97	98.41	–	–	1.99	1.35	0.10	2.00	1.97	99.98	–	–
	Area 2	0.51	0.24	1.91	1.00	1.56	95.36	–	–	1.99	2.00	0.68	1.45	1.99	98.52	–	–

**Table 8:** Dynamic responses of the system.

Controller	Optimization Method	∆f_1_			∆f_2_			∆P_tie_			V_out1_				V_out2_			
		MO_∆f1_ (Hz)	MU_∆f1_ (Hz)	ESS_∆f1_ (p.u)	MO_∆f2_ (Hz)	MU_∆f2_ (Hz)	ESS_∆f2_ (p.u)	MO_∆P_ (p.u)	MU_∆P_ (p.u)	ESS_∆P_ (p.u)	MP_-V1_ (p.u)	T_r-V1 (s)_	T_s-V1 (s)_	ESS_V1_ (p.u)	MP_-V2_ (p.u)	T_r-V2 (s)_	T_s-V2 (s)_	ESS_V2_ (p.u)
Initial FLC	PSO	0.004	− 0.020	0.0005	0.001	− 0.019	0.0005	0.0001	0.0000	0.0001	0.39	0.07	3.5	0.00003	0.3589	0.07	3.4	0.00003
Initial FLC	GTO	0.004	− 0.021	0.0005	0.001	− 0.012	0.0005	0.0002	− 0.0004	0.0001	0.36	0.07	3.6	0.00003	0.1358	0.20	2.9	0.00006
Initial FLC	MPA	0.007	− 0.024	0.0005	0.001	− 0.012	0.0005	0.0002	− 0.0005	0.0001	0.27	0.14	2.1	0.00003	0.1359	0.21	2.8	0.00006
Enhanced FLC	PSO	0.000	− 0.024	0.0000	0.017	− 0.041	0.0000	0.0004	− 0.0005	0.0001	0.52	0.08	3.8	0.00000	0.3459	0.07	7.5	0.00000
Enhanced FLC	GTO	0.011	− 0.082	0.0000	0.002	− 0.060	0.0000	0.0006	− 0.0013	0.0000	0.26	0.14	7.5	0.00000	0.5763	0.11	7.1	0.00000
Enhanced FLC	MPA	0.004	− 0.032	0.0000	0.000	− 0.014	0.0000	0.0002	− 0.0021	0.0000	0.38	0.26	3.8	0.00000	0.1931	0.16	1.8	0.00000

Table 8 presents a comparative analysis of the Initial and Enhanced FLCs by applying the three optimization algorithms, including PSO, GTO, and MPA. Based on the results obtained in the table, it has been identified that the Enhanced FLC has the ability to reach zero steady-state error, and achieve better dynamic regulation.

As for frequency deviations in both areas (Δf_1_ and Δf_2_), the Enhanced FLC eliminates steady-state errors in both areas reaching 0.0000 p.u. with all optimization algorithms, whereas the Initial FLC exhibits a small offset of 0.0005 p.u.

Regarding tie-line power deviations (ΔP_tie_), it is found that the Enhanced FLC provides better accuracy, as it achieves zero in steady-state errors, reaching 0.0000 p.u. when both GTO and MPA are used, compared to 0.0001 p.u. for the Initial FLC.

As for the voltage responses in both areas (Vout_1_ and Vout_2_) the Enhanced FLC further succeeds to eliminate the steady-state errors, unlike the Initial FLC, where small offsets were observed.

Overall, the Enhanced FLC, especially when optimized using the MPA, considerably improves steady-state accuracy and enhances damping, which confirms its robustness and effective control performance in multi-area power systems.

The corresponding ITAE values and computational times for all controllers and optimization algorithms are demonstrated in Table 9.

**Table 9:** Comparative ITAE and simulation runtime for the Initial and Enhanced FLCs.

Controller	Best ITAE value			Simulation runtime (minutes/optimization run)		
	PSO	GTO	MPA	PSO	GTO	MPA
Initial FLC	53.6	53.9	53.2	16	19	24
Enhanced FLC	20.1	11.5	11.1	2	3	4

The results illustrated in Table 7 generally indicate that the Enhanced FLC succeeded in achieving a remarkable performance improvement compared to the Initial design. Particularly, the Enhanced FLC succeeded in providing a significantly lower ITAE value compared to the Initial FLC under the three optimization algorithms. It is important to note that when the Enhanced FLC is tuned using the MPA algorithm, it achieves the best performance among all optimizers, with an ITAE value of 11.1. This value represents an improvement of approximately 80% compared to the Initial FLC controller design tuned using the same optimizer, with ITAE of 53.2. The GTO algorithm also provides almost similar results with ITAE of 11.5.

In terms of computational efficiency, the Enhanced FLC dramatically reduces simulation runtime per optimization run. The MPA-optimized Enhanced FLC requires only 4 min, compared with an average of 23 min for the Initial FLC. GTO and PSO achieve 3 and 2 min, compared to 19 and 16 min for the Initial FLC, respectively. This reduction implies that the proposed controller can be re-tuned much more rapidly, which is beneficial for scenarios requiring repeated optimization (e.g., adaptive control or system reconfiguration). It is important to note that these times refer exclusively to the simulation runtime of the optimization process; the online execution time of the controller (i.e., the time to compute one control output) is dominated by the fuzzy inference cycle (typically microseconds) and is nearly identical for both Initial and Enhanced FLCs, since both share the same rule base and membership function structure (only the crisp range values differ). Overall, the Enhanced FLC especially when tuned using MPA or GTO offers better accuracy, robustness, and real-time feasibility for multi-area power system control than the Initial FLC.

#### Results summary

In this case study, the Initial FLC controllers provided basic system stability, achieving a best ITAE value of nearly 53. In contrast, the Enhanced FLC demonstrates significant performance improvements, this is because it is able to eliminate the of steady-state error, show effective damping of the transient response, and provide smooth recovery, resulting in a best ITAE value of approximately 11. Particularly, the Enhanced FLC, which was tuned using the MPA and GTO, showed significantly improved results compared to the one tuned using the PSO algorithm.

As for computational time, the Enhanced FLC drastically reduced the computational load compared to the Initial FLC, completing each cycle in only 2–4 min, making it more suitable for real-time multi-area power system applications.

Such results indicate that the Enhanced FLC demonstrates significantly better performance when deployed to control multi-area power systems under step load perturbations. These improvements translate into Enhanced dynamic response, and greater robustness. In this context, MPA and GTO present the preferred optimizers.

### Case study 2: impact of renewable energy integration (photovoltaic and wind turbine)

#### System under study

This case study examines the two-area interconnected power system as illustrated in Fig. 1, where a PV unit is added to Area 1 at 20 s, and a wind turbine to Area 2 at 70 s. Sequential 2.5% load perturbations are applied at 50 s and 150 s to enable assessment of controller performance under combined renewable sources and disturbances.

#### Simulation results

**Fig. 9:**
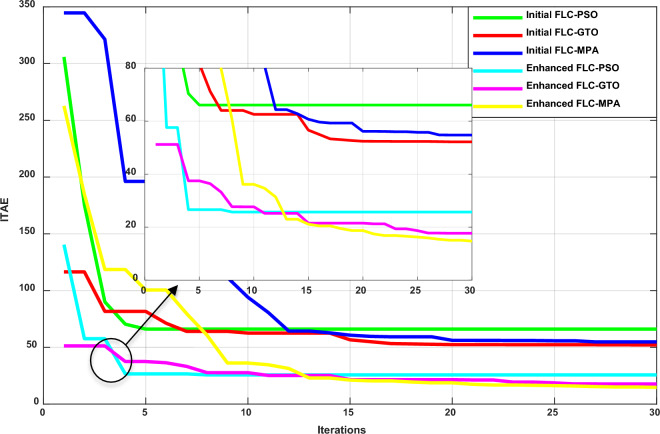
Convergence profile of the ITAE obtained during the optimization of controller parameters in the 2nd case study. The curves correspond to: Initial FLC–PSO (Green), Initial FLC–GTO (Red), Initial FLC–MPA (Blue), Enhanced FLC–PSO (Cyan), Enhanced FLC–GTO (Magenta), and Enhanced FLC–MPA (Yellow).

**Fig. 10:**
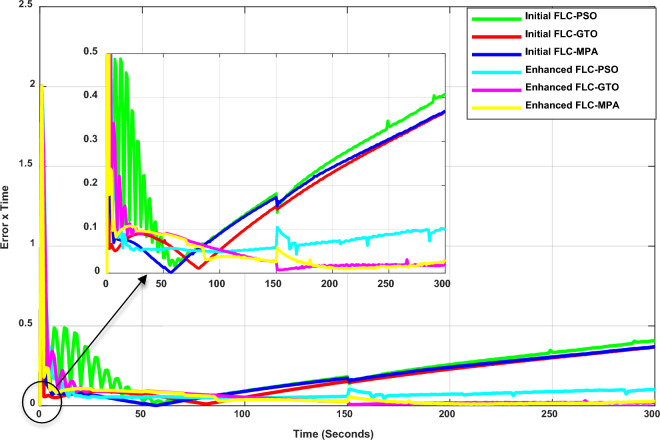
The ITAE Integrand (Error × Time) during controller performance evaluation in Case Study No.2. The curves correspond to: Initial FLC–PSO (Green), Initial FLC–GTO (Red), Initial FLC–MPA (Blue), Enhanced FLC–PSO (Cyan), Enhanced FLC–GTO (Magenta), and Enhanced FLC–MPA (Yellow).

**Fig. 11:**
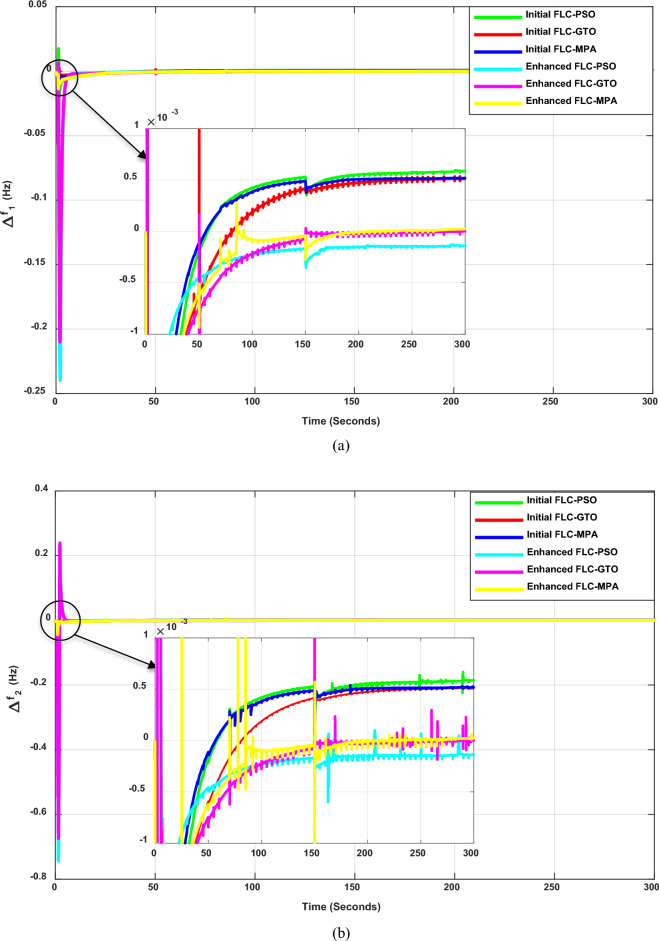

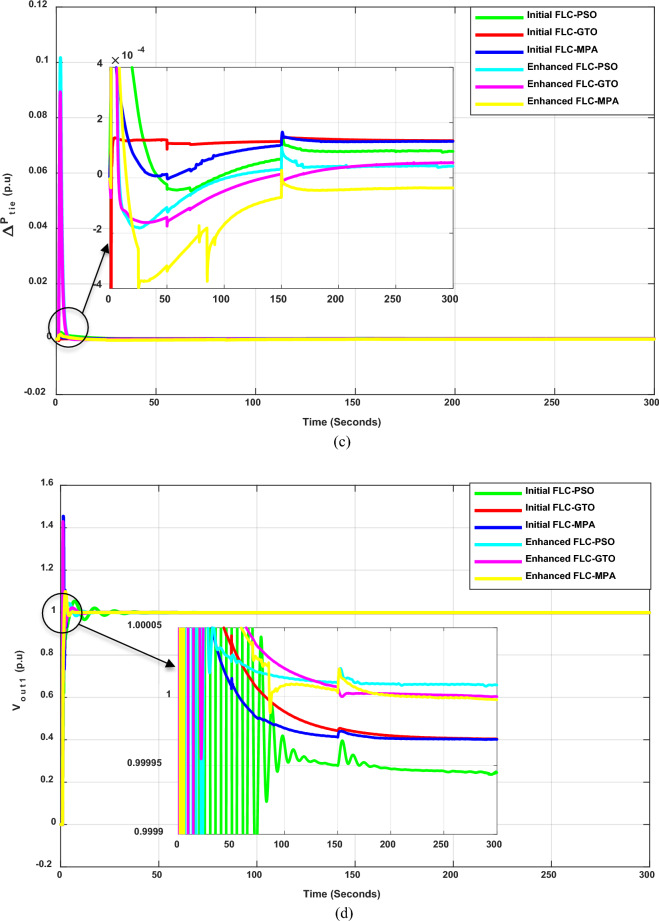

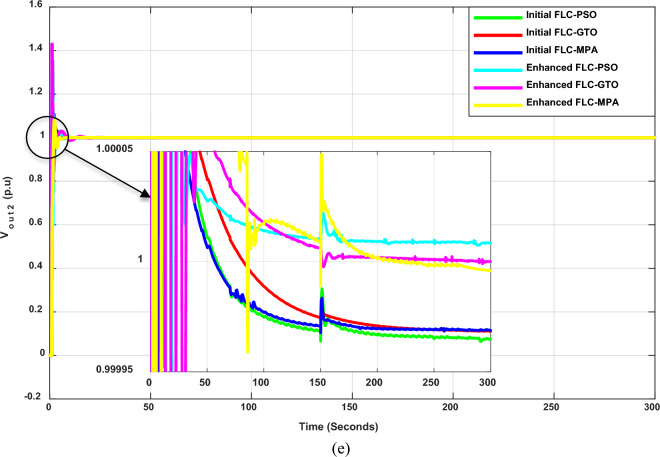
Time-domain responses of the two-area interconnected system under the 2nd case study: (**a**) ∆f_1_, (**b**) ∆f_2_, (**c**) ΔP_tie_, (**d**) V_out1_, (**e**) V_out2_. The curves correspond to: Initial FLC–PSO (Green), Initial FLC–GTO (Red), Initial FLC–MPA (Blue), Enhanced FLC–PSO (Cyan), Enhanced FLC–GTO (Magenta), and Enhanced FLC–MPA (Yellow).

Figure 9 illustrates the convergence profiles of the Integral Time Absolute Error (ITAE), while the corresponding time–error (Error × Time) is depicted in Fig. 10. The dynamic behavior of the power system is presented in Fig. 11.

As shown in Fig. 9, all Initial FLC controllers optimized by the three optimization algorithms, including PSO, GTO, or MPA, have a convergence slow rate and high ITAE values.

On the contrary, the proposed Enhanced FLC structure is able to attain a substantial reduction in the ITAE and a convergence faster rate under all the optimization techniques compared to the Initial ITAE. In addition, the proposed Enhanced FLC structure optimized by the MPA and GTO optimizers yields the best performance, as it rapidly converges to the final ITAE values of 15 and 18, respectively. Moreover, the proposed Enhanced FLC optimized by the PSO optimizer also attains a substantial improvement compared to the Initial FLC, as it converges to the ITAE of 25.

Overall, the results confirm performance improvement provided by the Enhanced FLC architecture in convergence characteristics and system stability.

Figure 10 shows the error–time performance curve for the Initial FLC and the Enhanced FLC. With respect to the Initial FLC under all optimization algorithms, it shows slow convergence rates, resulting in a gradual increase in error over time.

On the other hand, the Enhanced FLC optimized using the three optimization algorithms, demonstrates better performance compared to the Initial FLC. In particular, the Enhanced FLC using the GTO and MPA optimization algorithms showed rapid convergence to an error level close to zero, along with the lowest level of the steady-state error. Even though the Enhanced FLC using the PSO algorithm showed an increase in error over time, its performance was significantly higher than all of the Initial FLC configurations.

To summarize, these results clearly confirm that the Enhanced FLC architecture delivers improved dynamic performance compared with the Initial FLC configuration. Moreover, both of GTO and MPA optimization algorithms demonstrate greater robustness than PSO.

As shown in Fig. 11, the Enhanced FLC controllers exhibit improved dynamic performance compared with the Initial FLC designs, as clearly reflected in the responses of the key system parameters, including frequency and tie-line power deviations, and voltage regulation. As for frequency deviations (Δf_1_ and Δf_2_), the Enhanced controllers achieve negligible steady-state error. Regarding tie-line power deviations (ΔP_tie_), the responses are smoother with the Enhanced FLC, particularly for MPA and GTO variants. With respect to Voltage responses (Vout_1_ and Vout_2_), they also demonstrate rapid recovery under the Enhanced controllers. To summarize, the Enhanced FLC framework provides improved robustness, Enhanced stability, and stronger disturbance rejection capability, particularly when tuned using the MPA and GTO optimizers.

The optimized parameter values identified for the 2nd case study are summarized in Tables 10 and 11, while the corresponding time-domain dynamic performance indicators of the power system are reported in Table 12.

**Table 10:** Optimized tuning parameters of the LFC controllers for both interconnected areas.

Optimizer	Area	Initial FLC								Enhanced FLC							
		K_P_	K_I_	K_D1_	K_D2_	N_1_	N_2_	K_E_	K_CE_	K_P_	K_I_	K_D1_	K_D2_	N_1_	N_2_	K_E_	K_CE_
PSO	Area 1	100.0	2.00	1.02	6.35	1.98	100.0	2.00	0.07	76.44	1.82	2.00	10.00	1.60	64.20	2.00	0.10
	Area 2	43.98	2.00	2.00	9.57	0.67	75.36	1.13	0.07	25.98	2.00	1.09	4.68	2.00	95.84	2.00	0.10
GTO	Area 1	100.0	2.00	0.73	7.06	2.00	0.10	2.00	0.10	100.0	1.67	0.98	1.37	0.60	99.97	1.72	0.01
	Area 2	100.0	2.00	2.00	10.00	2.00	100.0	2.00	0.01	54.26	2.00	0.45	2.15	0.27	99.96	2.00	0.01
MPA	Area 1	80.10	2.00	2.00	9.94	2.00	99.96	2.00	0.06	91.46	1.80	1.98	4.52	0.87	99.99	1.99	0.08
	Area 2	44.78	2.00	1.99	10.00	0.36	99.05	1.98	0.09	25.93	1.69	0.33	5.11	1.89	48.15	2.00	0.01

**Table 11:** Optimized tuning parameters of the AVR controllers for both interconnected areas.

Optimizer	Area	Initial FLC								Enhanced FLC							
		K_P_	K_I_	K_D1_	K_D2_	N_1_	N_2_	K_E_	K_CE_	K_P_	K_I_	K_D1_	K_D2_	N_1_	N_2_	K_E_	K_CE_
PSO	Area 1	0.97	1.26	2.00	2.00	1.25	0.1	–	–	1.25	2.00	1.06	2.00	2.00	100.0	–	–
	Area 2	1.45	2.00	1.04	2.00	1.20	100.0	–	–	2.00	2.00	2.00	2.00	2.00	100.0	–	–
GTO	Area 1	2.00	2.00	0.77	1.09	2.00	0.1	–	–	1.52	1.31	1.89	1.37	1.43	100.0	–	–
	Area 2	1.92	1.95	1.05	0.72	1.40	100.0	–	–	1.18	1.55	1.51	2.00	1.85	99.98	–	–
MPA	Area 1	1.97	1.99	1.98	0.21	2.00	77.44	–	–	1.06	1.18	1.98	0.33	0.15	59.92	–	–
	Area 2	0.75	2.00	1.35	0.60	2.00	14.92	–	–	1.26	0.65	1.24	0.69	0.12	97.27	–	–

**Table 12:** Dynamic responses of the system.

Controller	Optimization Method	∆f_1_			∆f_2_			∆P_tie_			V_out1_				V_out2_			
		MO_∆f1_ (Hz)	MU_∆f1_ (Hz)	ESS_∆f1_ (p.u)	MO_∆f2_ (Hz)	MU_∆f2_ (Hz)	ESS_∆f2_ (p.u)	MO_∆P_ (p.u)	MU_∆P_ (p.u)	ESS_∆P_ (p.u)	MP_-V1_ (p.u)	T_r-V1 (s)_	T_s-V1 (s)_	ESS_V1_ (p.u)	MP_-V2_ (p.u)	T_r-V2 (s)_	T_s-V2 (s)_	ESS_V2_ (p.u)
Initial FLC	PSO	0.017	− 0.041	0.0006	0.001	− 0.031	0.0006	0.0025	− 0.0002	0.0001	0.31	0.07	17.8	0.00006	0.1534	0.15	4.3	0.00004
Initial FLC	GTO	0.003	− 0.022	0.0005	0.001	− 0.012	0.0005	0.0001	− 0.0005	0.0001	0.18	0.17	1.8	0.00003	0.1913	0.19	1.9	0.00003
Initial FLC	MPA	0.001	− 0.014	0.0005	0.001	− 0.023	0.0005	0.0012	− 0.0000	0.0001	0.45	0.19	3.3	0.00003	0.3550	0.18	4.1	0.00003
Enhanced FLC	PSO	0.000	− 0.240	0.0001	0.234	− 0.744	0.0001	0.1017	− 0.0002	0.0000	0.34	0.12	5.7	0.00001	0.3572	0.07	3.3	0.00001
Enhanced FLC	GTO	0.006	− 0.210	0.0000	0.239	− 0.674	0.0000	0.0893	− 0.0002	0.0001	0.43	0.10	6.5	0.00000	0.4296	0.09	7.1	0.00000
Enhanced FLC	MPA	0.000	− 0.014	0.0000	0.005	− 0.044	0.0000	0.0017	− 0.0004	0.0000	0.10	0.59	3.4	0.00000	0.0820	0.68	4.3	0.00000

Table 12 shows a comparison of the dynamic performance of both Enhanced and Initial FLCs, employing all three optimization methods, namely PSO, GTO, and MPA. The results reveal a clear trade-off between steady-state accuracy and transient peak magnitudes.

As presented, the Enhanced FLC reduces steady-state error to nearly zero in frequency deviations (Δf_1_ and Δf) compared to the Initial FLC. Furthermore, the Enhanced FLC demonstrates improved transient response compared to the Initial FLC, in particular the FLC employing the MPA optimization method provides the best overall performance.

As for the tie-line power deviations (ΔP_tie_), the Enhanced FLC demonstrates better damping characteristics, and improved steady-state accuracy than the Initial FLC. This is particularly true both the MPA and GTO optimization methods are involved.

With respect to voltage response (Vout_1_, Vout_2_), it is found that the Enhanced FLC has better performance characteristics than the Initial FLC. This is because the steady-state error is zero in this case, unlike in the Initial FLC where there is a slight deviation.

However, some Enhanced FLC variants exhibit larger transient peaks compared to their Initial counterparts. For example:

- With GTO, the Enhanced FLC shows larger frequency undershoots (MU_∆f1_ = − 0.210 Hz vs. − 0.022 Hz for Initial FLC-GTO) and a substantially larger tie-line overshoot (MO_∆P_ = 0.0893 p.u. vs. 0.0001 p.u.).
- With PSO, the Enhanced FLC also exhibits larger undershoots (MU_∆f1_ = − 0.240 Hz vs. − 0.041 Hz) and a much higher tie-line overshoot (0.1017 p.u. vs. 0.0025 p.u.).
- In contrast, the MPA-tuned Enhanced FLC achieves the best balance: near-zero steady-state error with only modest increases in transient peaks (MU_∆f1_ = − 0.014 Hz, MO_∆P_ = 0.0017 p.u.).

This trade-off between transient peaks and steady-state performance is often acceptable, particularly when long-term regulation under renewable intermittency is the primary objective. The seemingly contradictory behavior, where some Enhanced FLC variants exhibit larger transient peaks yet achieve superior steady-state accuracy, is a direct consequence of the ITAE objective function. Since ITAE penalizes errors that persist over time more heavily than short-lived deviations, the optimizer permits higher initial overshoots if they lead to faster long-term error reduction.

The Enhanced FLC, by shifting the zero-output crisp range rightward, introduces a slight positive bias that accelerates error correction following disturbances, albeit at the cost of increased initial overshoot. In contrast, the Initial FLC employs a symmetric zero region, resulting in smaller transient peaks but slower error decay. This trade-off is clearly reflected in Table 23, where significant improvements in steady-state error are accompanied by occasional increases in rise and settling times.

To sum up, the Enhanced FLC provides superior steady-state accuracy and damping of residual oscillations for all optimizers, but transient peaks may increase for PSO and GTO variants. The MPA-tuned Enhanced FLC offers the most balanced performance. The Corresponding ITAE scores as well as simulation runtimes are depicted in Table 13.

**Table 13:** Comparative ITAE and simulation runtime for the Initial and Enhanced FLCs.

Controller	Best ITAE value			Simulation runtime (minutes/optimization run)		
	PSO	GTO	MPA	PSO	GTO	MPA
Initial FLC	66.1	52.2	54.8	16	19	25
Enhanced FLC	25.7	17.7	14.7	3	4	6

The data presented in Table 13 clearly highlight the advantages offered by the Enhanced FLC over the Initial FLC in terms of accuracy and execution speed. As for the ITAE index, the Initial FLC controller provides high values of integrated errors, with the best value of almost 52 obtained using the GTO method, which could be considered a predictable outcome for slow convergence and poor disturbance rejection capability. In contrast, the Enhanced FLC significantly reduces ITAE values, regardless of the optimizer used. The MPA-tuned Enhanced FLC demonstrates the best performance with an ITAE of 15, reducing the best result of the Initial model by nearly three-quarters. The GTO-Enhanced model takes the next position with an ITAE of 18, while even the performance of the PSO model demonstrates better performance compared to all results of the Initial FLC with an ITAE of 26.

Regarding computational efficiency, the Enhanced FLC attains much smaller simulation runtimes compared to the Initial FLC, namely the Initial FLC. For example, the MPA-optimized Enhanced controller takes only 6 min to complete an operational cycle, while the Initial FLC takes as much as 25 min. Similarly, using PSO and GTO optimization, the Enhanced FLC completes the cycle in 3 and 4 min, respectively, whereas the Initial FLC requires 16 and 19 min.

Overall, the Enhanced FLC, particularly when tuned using the MPA or GTO techniques, provides better performance in terms of achieving an effective balance between accuracy, speed, and robustness compared to the Initial FLC configuration.

#### Results summary

The results obtained through this case study reveal that the Initial FLC Controllers have the capability to achieve system stability. However, the FLC Controllers have been greatly affected by the presence of renewable energy sources, resulting in poor performance in terms of ITAE scores, indicating control precision and disturbances mitigation. On the other hand, the Enhanced FLC model has been able to achieve significant improvements in the overall performance, where the model has been able to achieve virtually zero steady-state deviations, along with faster damping, and smoother dynamic responses.

Among the tested approaches, the Enhanced FLC tuned using MPA provides the best overall performance, offering an optimal balance between transient behavior and steady-state accuracy. Although the Enhanced FLC tuned with PSO and GTO eliminates steady-state error, it produces larger frequency undershoots and tie-line overshoots compared to the Initial FLC, as shown in Table 10.

Furthermore, the Enhanced FLC significantly reduces simulation runtime, requiring approximately one-fifth of the computational effort of the Initial FLC. Overall, for managing key variables in multi-area systems with renewable penetration, including frequency, voltage, and tie-line power, the Enhanced FLC architecture outperforms the Initial design due to its superior disturbance rejection and precise long-term regulation.

These results are supported by theoretical insights. The superior performance of MPA and GTO compared to PSO is attributed to their stronger ability to avoid premature convergence in non-convex, multi-modal search spaces. The trade-off between transient peaks and steady-state accuracy arises from the ITAE objective, which emphasizes long-term error reduction. Additionally, the effectiveness of shifting the zero-output crisp range is explained by compensation of actuator and sensor asymmetries and an increase in local gain near equilibrium, while excessive shifting can introduce steady-state error of opposite sign. These interpretations provide control-theoretic justification for the observed results.

### Case study 3: sensitivity assessment of system parameters

#### System under study

This case study is based on the power system model used in Case Study No. 2, with certain modifications applied to specific power system parameters. Specifically, critical model constants, such as time constants, controller gains, and droop coefficients, are systematically altered to investigate the effect of system configuration variations on controller response and dynamic behavior under operating conditions comparable to the preceding case. The selected parameter adjustments are the same as those applied to our previous study^71^ to guarantee a consistent comparison.

#### Simulation results

**Fig. 12:**
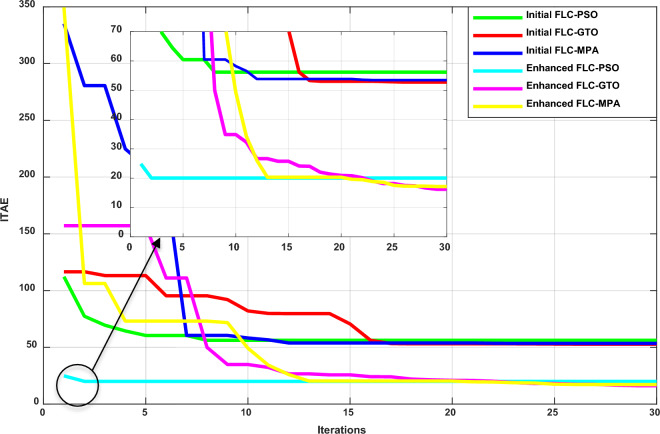
Convergence profile of the ITAE obtained during the optimization of controller parameters in the 3rd case study. The curves correspond to: Initial FLC–PSO (Green), Initial FLC–GTO (Red), Initial FLC–MPA (Blue), Enhanced FLC–PSO (Cyan), Enhanced FLC–GTO (Magenta), and Enhanced FLC–MPA (Yellow).

**Fig. 13:**
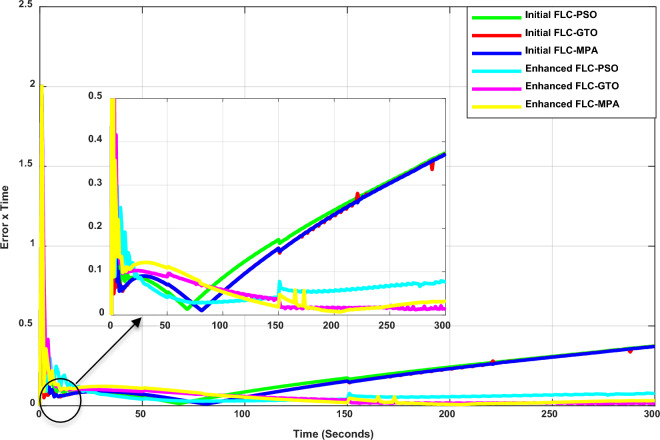
The ITAE Integrand (Error × Time) during controller performance evaluation in Case Study No.3. The curves correspond to: Initial FLC–PSO (Green), Initial FLC–GTO (Red), Initial FLC–MPA (Blue), Enhanced FLC–PSO (Cyan), Enhanced FLC–GTO (Magenta), and Enhanced FLC–MPA (Yellow).

**Fig. 14:**
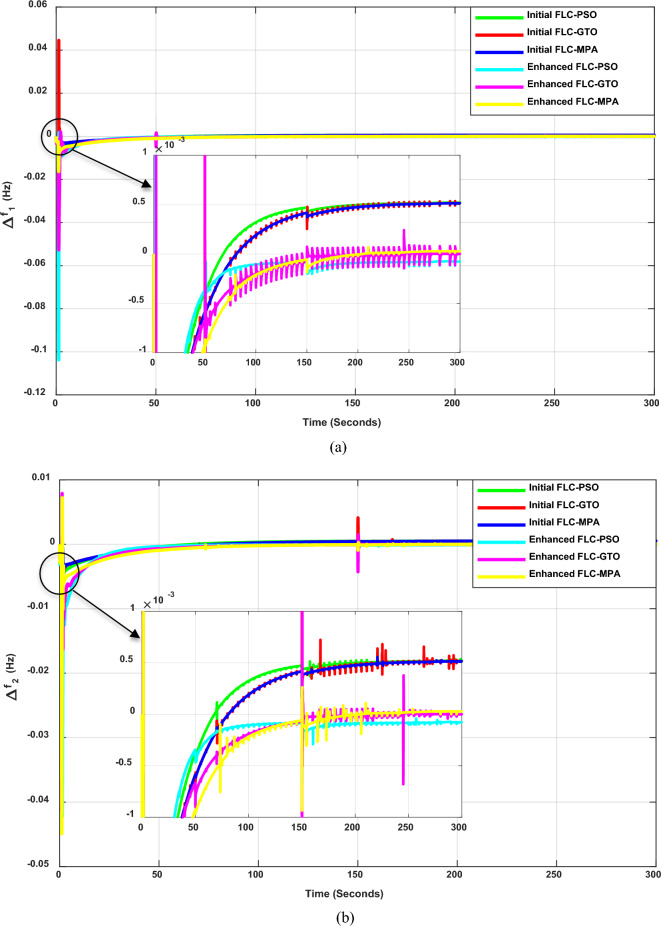

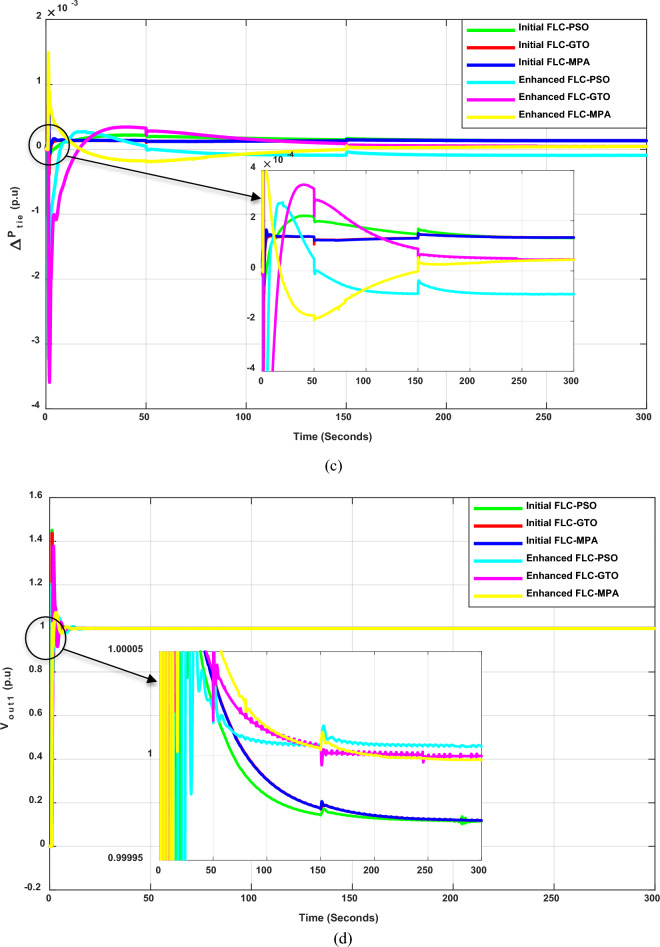

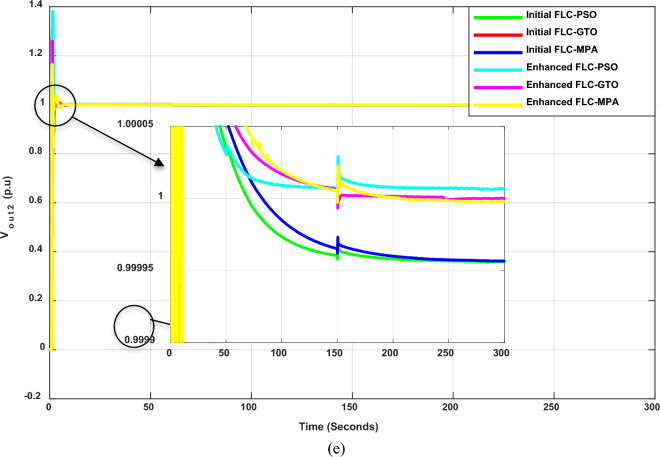
Time-domain responses of the two-area interconnected system under the 3rd case study: (**a**) ∆f_1_, (**b**) ∆f_2_, (**c**) ΔP_tie_, (**d**) V_out1_, (**e**) V_out2_. The curves correspond to: Initial FLC–PSO (Green), Initial FLC–GTO (Red), Initial FLC–MPA (Blue), Enhanced FLC–PSO (Cyan), Enhanced FLC–GTO (Magenta), and Enhanced FLC–MPA (Yellow).

Figure 12 displays the convergence curves of the Integral Time Absolute Error (ITAE), while the integrand of the above convergence curves (Error × Time), is depicted in Fig. 13. In addition, the dynamic responses of the power system are illustrated in Fig. 14.

As can be observed in Fig. 12, the results of the three Initial FLC designs tuned using the three optimizers are almost the same, as they all converge to a final ITAE value above 50. On the other hand, the results obtained from the Enhanced FLC design have lower ITAE scores compared to the Initial controller. Both the GTO and MPA optimized versions demonstrate the most effective performance, achieving rapid convergence to a value near 10. Although the PSO-tuned version ranks last, with an ITAE value of 20, it still represents a significant improvement compared to the Initial controllers.

As presented in Fig. 13, the error dynamics (Error × Time) for the evaluated controllers of the Initial FLC designs converge slowly and exhibit a higher steady-state residual error, regardless of the tuning algorithm. In contrast, the Enhanced FLC demonstrates significant improvement in transient and steady-state behavior compared to the Initial FLC.

As illustrated in Fig. 14, the two-area power system’s dynamic responses demonstrate clear limitations of the Initial FLC controllers. Regarding, both frequency deviation signals (Δf_1_ and Δf_2_), Initial FLC demonstrates insufficient damping performance regardless of the optimization algorithms. In contrast, the dynamic performance of the Enhanced FLC controllers is significantly better with negligible steady-state error.

As for the tie line power deviations (ΔP_tie_), the Initial FLC controllers exhibits slow convergence, and so poor disturbance rejection and interarea coordination. In contrast, the performance of the Enhanced FLC design shows better stability, as indicated by the smallest steady-state deviation, particularly when optimized using MPA. The GTO tuning version also shows almost similar performance characteristics. In addition, the PSO tuning version of the Enhanced FLC controller, although having a relatively higher value of the Initial overshoot, it achieves improved performance in comparison with the Initial FLC controllers.

As for voltage regulation, the difference between the configurations of the two controllers, namely Initial FLC and Enhanced FLC, is again highlighted. Although Initial FLC permit substantial voltage sags accompanied by long transients. Enhanced FLC controllers generally provide rapid recovery, strong voltage regulation, and smooth settling. While MPA and GTO demonstrate the strongest overall performance, it should be noted that the PSO-tuned controller also performs significantly better than any of the Initial controllers.

In conclusion, it may be said that the performance of the Enhanced FLC demonstrates better performance compared to that of the Initial FLC, regardless of whether it is in frequency regulation, tie-line power stability, or voltage regulation. The MPA and GTO deployed to tune FLC stand out for their stronger damping, better dynamic performance, and dependability.

The optimized parameter values identified for the 3rd case study are summarized in Tables 14 and 15, while the corresponding time-domain dynamic performance indicators of the power system are reported in Table 16.

**Table 14:** Optimized tuning parameters of the LFC controllers for both interconnected areas.

Optimizer	Area	Initial FLC								Enhanced FLC							
		K_P_	K_I_	K_D1_	K_D2_	N_1_	N_2_	K_E_	K_CE_	K_P_	K_I_	K_D1_	K_D2_	N_1_	N_2_	K_E_	K_CE_
PSO	Area 1	65.46	2.00	2.00	9.95	1.80	100.0	2.00	0.09	22.92	2.00	0.93	5.39	1.82	50.33	1.77	0.05
	Area 2	91.58	2.00	1.61	7.70	2.00	54.50	1.89	0.08	40.05	1.69	1.79	5.43	1.67	74.49	1.63	0.03
GTO	Area 1	100.0	2.00	0.13	1.27	2.00	100.0	2.00	0.10	25.73	1.70	0.32	0.96	0.21	93.34	1.72	0.01
	Area 2	100.0	2.00	2.00	0.10	2.00	100.0	2.00	0.10	100.0	1.69	0.37	0.10	2.00	100.0	2.00	0.01
MPA	Area 1	100.0	2.00	2.00	8.39	1.98	46.96	2.00	0.07	94.21	1.65	1.83	4.72	1.63	99.71	2.00	0.10
	Area 2	99.48	2.00	2.00	4.77	1.73	98.20	2.00	0.10	45.22	1.82	0.82	4.18	0.42	54.96	1.93	0.04

**Table 15:** Optimized tuning parameters of the AVR controllers for both interconnected areas.

Optimizer	Area	Initial FLC								Enhanced FLC							
		K_P_	K_I_	K_D1_	K_D2_	N_1_	N_2_	K_E_	K_CE_	K_P_	K_I_	K_D1_	K_D2_	N_1_	N_2_	K_E_	K_CE_
PSO	Area 1	2.00	2.00	1.53	2.00	1.56	100.0	–	–	0.48	1.98	1.84	0.87	1.41	65.47	–	–
	Area 2	2.00	1.81	1.15	2.00	1.70	100.0	–	–	1.86	0.31	1.13	2.00	0.90	100.0	–	–
GTO	Area 1	2.00	2.00	2.00	1.10	1.98	96.17	–	–	0.53	2.00	0.16	2.00	2.00	4.76	–	–
	Area 2	0.10	0.13	2.00	1.72	1.31	100.0	–	–	0.39	1.41	2.00	2.00	1.21	93.73	–	–
MPA	Area 1	0.72	2.00	2.00	1.37	0.18	0.10	–	–	0.63	1.82	1.50	0.43	0.69	68.15	–	–
	Area 2	1.99	2.00	0.87	1.02	1.53	60.49	–	–	1.94	1.03	1.84	2.00	0.53	93.71	–	–

**Table 16:** Dynamic responses of the system.

Controller	Optimization Method	∆f_1_			∆f_2_			∆P_tie_			V_out1_				V_out2_			
		MO_∆f1_ (Hz)	MU_∆f1_ (Hz)	ESS_∆f1_ (p.u)	MO_∆f2_ (Hz)	MU_∆f2_ (Hz)	ESS_∆f2_ (p.u)	MO_∆P_ (p.u)	MU_∆P_ (p.u)	ESS_∆P_ (p.u)	MP_-V1_ (p.u)	T_r-V1 (s)_	T_s-V1 (s)_	ESS_V1_ (p.u)	MP_-V2_ (p.u)	T_r-V2 (s)_	T_s-V2 (s)_	ESS_V2_ (p.u)
Initial FLC	PSO	0.001	− 0.018	0.0005	0.001	− 0.020	0.0005	0.0003	− 0.0001	0.0001	0.45	0.08	5.6	0.00003	0.2921	0.15	2.1	0.00004
Initial FLC	GTO	0.045	− 0.054	0.0005	0.004	− 0.034	0.0005	0.0001	− 0.0011	0.0001	0.44	0.11	2.7	0.00003	0.2738	0.15	2.1	0.00004
Initial FLC	MPA	0.001	− 0.010	0.0005	0.001	− 0.015	0.0005	0.0006	− 0.0001	0.0001	0.34	0.30	4.7	0.00003	0.3131	0.18	2.2	0.00004
Enhanced FLC	PSO	0.002	− 0.104	0.0001	0.000	− 0.042	0.0001	0.0003	− 0.0032	0.0001	0.21	0.14	5.8	0.00000	0.3806	0.22	2.8	0.00001
Enhanced FLC	GTO	0.002	− 0.053	0.0000	0.008	− 0.041	0.0000	0.0007	− 0.0036	0.0000	0.38	0.33	6.0	0.00000	0.2588	0.14	2.1	0.00000
Enhanced FLC	MPA	0.000	− 0.016	0.0000	0.007	− 0.045	0.0000	0.0015	− 0.0002	0.0000	0.07	0.24	6.2	0.00000	0.1645	0.14	3.7	0.00000

A performance comparison between the Enhanced FLC and the conventional Initial FLC design tuned using PSO, GTO, and MPA optimization algorithms appears in Table 16. The results again demonstrate a trade-off, as the Enhanced FLC eliminates steady-state error for all metrics with MPA and GTO, and near-zero for PSO), but some transient peaks are larger than those of the Initial FLC.

For frequency regulation, the Enhanced FLC achieves zero steady-state error across all optimizers. However, with PSO and GTO, the frequency undershoots are noticeably larger than those of the Initial FLC. For example, the undershoot reaches –0.104 Hz for Enhanced PSO compared to –0.018 Hz for Initial PSO, while Enhanced GTO shows a value comparable to the Initial case. The MPA-based Enhanced FLC provides the best compromise, achieving zero steady-state error with the smallest undershoot of –0.016 Hz.

In terms of tie-line power, the Enhanced FLC with GTO and MPA eliminates steady-state error but introduces higher peak overshoots. The overshoot increases to 0.0007 p.u. for Enhanced GTO and 0.0015 p.u. for Enhanced MPA, compared to lower values in the Initial FLC. The PSO-based Enhanced FLC shows comparable overshoot to the Initial design.

For voltage response, all Enhanced FLC variants eliminate steady-state error. However, some cases exhibit longer settling times, such as 6.0 s for Enhanced GTO compared to 2.7 s for Initial GTO. This behavior aligns with the ITAE objective, which prioritizes long-term error reduction over transient speed.

Overall, the Enhanced FLC, particularly with MPA, provides the best steady-state accuracy and acceptable transient peaks. The trade-offs for PSO and GTO variants (larger undershoots, higher tie-line overshoot, longer settling times) are clearly visible in Table 15 and are discussed above.

The best ITAE values of all optimizers used to tune both Initial and Enhanced FLC and their computational times are summarized in Table 17.

**Table 17:** Comparative ITAE and simulation runtime for the Initial and Enhanced FLCs.

Controller	Best ITAE value			Simulation runtime (minutes / optimization run)		
	PSO	GTO	MPA	PSO	GTO	MPA
Initial FLC	56.2	52.8	53.5	17	22	28
Enhanced FLC	20.0	16.2	17.2	3	3	5

As can be observed in Table 17, the overall performance of the Enhanced FLC is better than the Initial FLC with all three optimizers, in terms of both ITAE values and average simulation runtimes.

As for the ITAE values, the Initial FLC configuration shows significantly higher ITAE values, with all greater than 50.

On the other hand, the Enhanced FLC configuration shows significantly reduced ITAE values. Of the three optimizers, GTO shows the best performance in terms of ITAE values, with a value of around 16, followed by MPA. Even PSO showed the poorest performance among the three optimizers, yet better than the Initial FLC. This shows an average improvement of around 69% compared to the best result obtained with the Initial FLC, which was nearly 53.

With regard to computational time, the Enhanced FLC was able to reduce average simulation runtime to nearly 4 min per control cycle, compared to 22 min per control cycle of the Initial FLC, representing around 80% reduction.

To conclude, the Enhanced FLC significantly improves control accuracy and real‑time applicability.

#### Results summary

The results of the sensitivity analysis have demonstrated that the Enhanced FLC controller performs significantly better than the Initial FLC controller. As for the Initial FLC, it does exhibit base level stability but with high ITAE scores. In contrast, the Enhanced FLC controller has demonstrated a significant improvement in all performance metrics. Accordingly, it is able to completely eliminate steady-state error not only for frequency, but also for tie-line power and voltage. In addition, the Enhanced performance is accompanied by an increase in the transient damping and decrease in the volatility of the recovery behavior.

A similar trend is observed in computational efficiency, as the Enhanced FLC achieves an average simulation runtime that is approximately 80% lower than that of the Initial FLC.

The optimization study also indicates that PSO, GTO, and MPA have almost similar results when they are applied for tuning the Initial FLC. However, the Enhanced FLC has already shown that MPA and GTO are capable of performing better than PSO when applied for tuning more sophisticated controllers, as demonstrated in this case study.

To sum up, the results of this case study demonstrate that the Enhanced FLC is a robust and effective high-performance controller for load frequency and voltage regulation within multi-area electric power systems with variability in parameters. In addition, both MPA and GTO have been identified as the top-performing optimization techniques deployed to tune the Enhanced FLC.

### Case study No.4: impact of random load variations

#### System under study

**Fig. 15:**
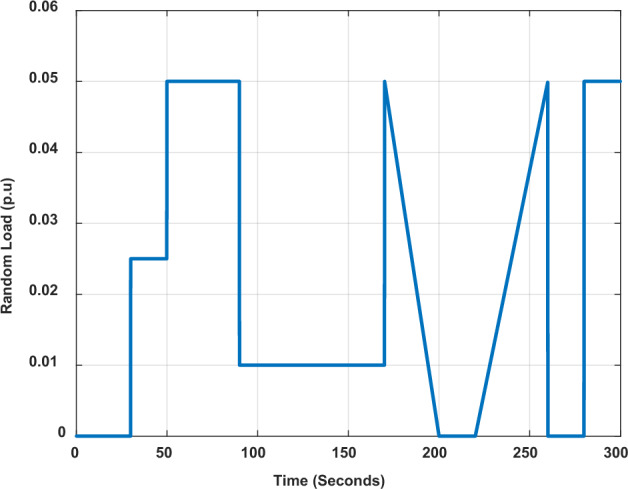
Input signal representing a random load disturbance (RLP) injected into Area-1 during Case Study 4.

The configuration of this case study mirrors that of Case Study No. 2, with only one key modification. This modification is applied only to Area-1 to be subjected to a random load perturbation (RLP), as depicted in Fig. 15. This change is introduced to evaluate controller performance under conditions of unexpected (random) load fluctuation.

#### Simulation results

**Fig. 16:**
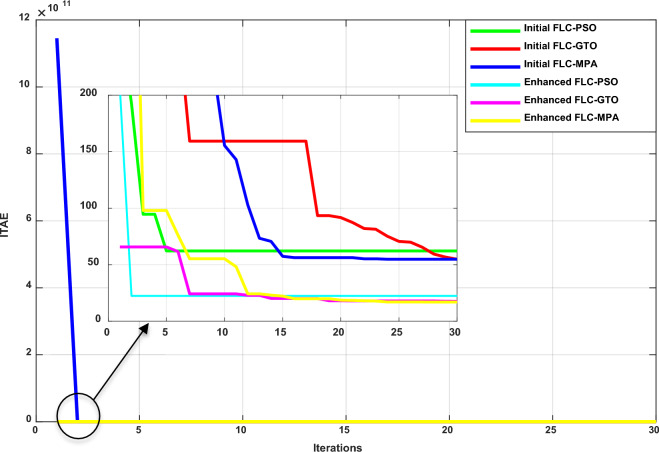
Convergence profile of the ITAE obtained during the optimization of controller parameters in the 4th case study. The curves correspond to: Initial FLC–PSO (Green), Initial FLC–GTO (Red), Initial FLC–MPA (Blue), Enhanced FLC–PSO (Cyan), Enhanced FLC–GTO (Magenta), and Enhanced FLC–MPA (Yellow).

**Fig. 17:**
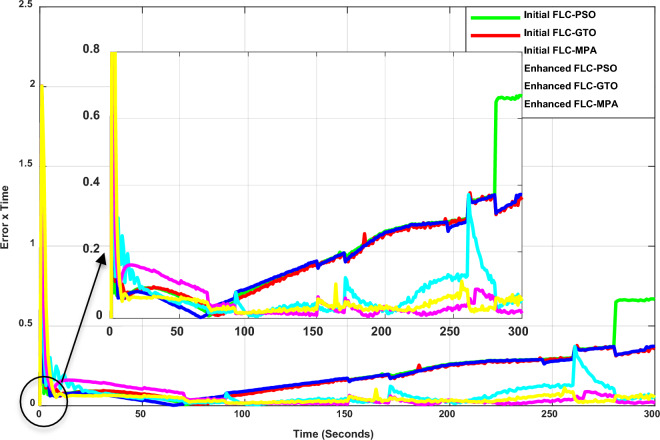
The ITAE Integrand (Error × Time) during controller performance evaluation in Case Study No.4. The curves correspond to: Initial FLC–PSO (Green), Initial FLC–GTO (Red), Initial FLC–MPA (Blue), Enhanced FLC–PSO (Cyan), Enhanced FLC–GTO (Magenta), and Enhanced FLC–MPA (Yellow).

**Fig. 18:**
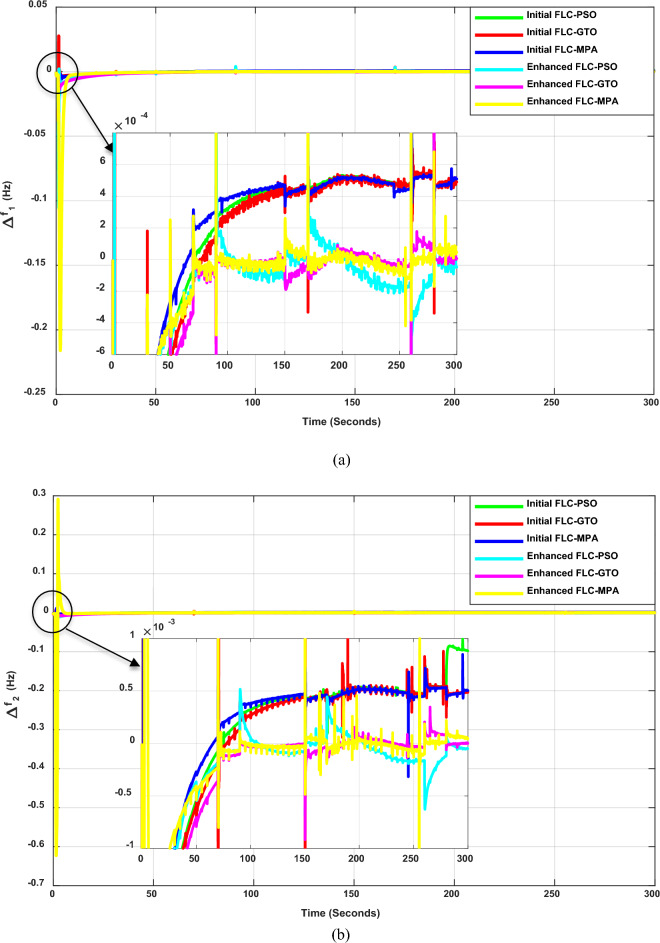

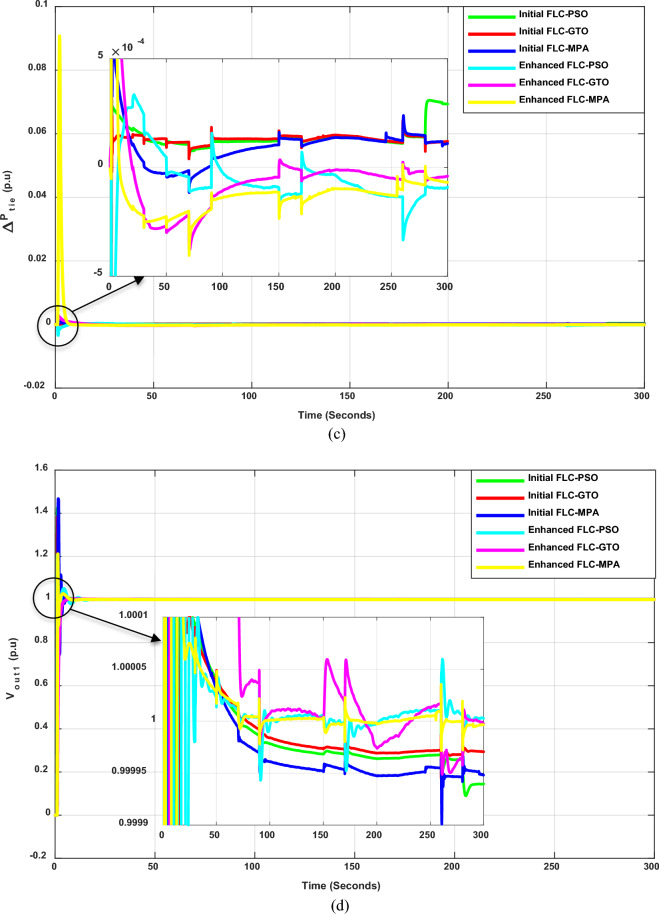

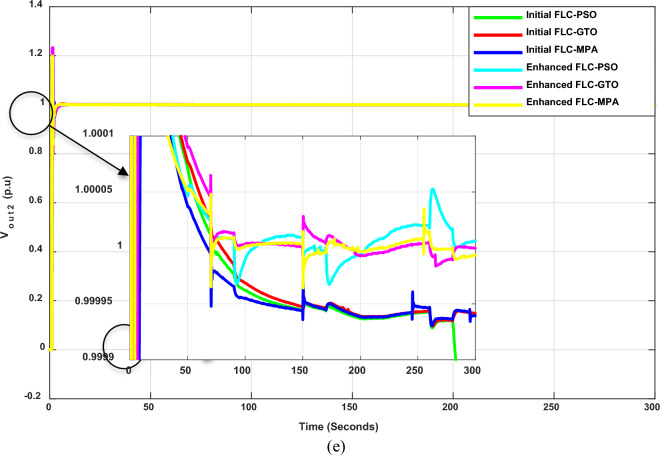
Time-domain responses of the two-area interconnected system under the 4th case study: (**a**) ∆f_1_, (**b**) ∆f_2_, (**c**) ΔP_tie_, (**d**) V_out1_, (**e**) V_out2_. The curves correspond to: Initial FLC–PSO (Green), Initial FLC–GTO (Red), Initial FLC–MPA (Blue), Enhanced FLC–PSO (Cyan), Enhanced FLC–GTO (Magenta), and Enhanced FLC–MPA (Yellow).

Figure 16 presents the convergence curves of the Integral Time Absolute Error (ITAE), while its integrand (Error × Time) is illustrated in Fig. 17. Figure 18 presents the dynamic responses of the power system.

As Fig. 16 depicts, it is clear that the Initial FLC optimized by the three optimizers, including PSO, GTO, and MPA, behaves almost identically. In addition, all of the Initial controllers converge to almost similar values of the ITAE performance index, which is around 60 regardless of the optimization technique employed. On the contrary, the Enhanced controllers show a considerable improvement in the performance by reducing the values of the ITAE performance index significantly compared to the Initial controllers. Specifically, the Enhanced controllers optimized by the GTO and MPA optimizers achieve the highest convergence rates and the lowest values of the ITAE performance index, which are close to 20. Although the performance of the PSO-Enhanced controller is considerably better than that of the Initial controllers, it is slightly worse than that of the other two Enhanced controllers.

The results for the time domain error response for both Initial and Enhanced controllers tuned using the three optimizers (PSO, GTO, and MPA) are presented in Fig. 17. The Initial FLC-based controllers are seen to have a slow convergence and large steady-state error. In addition, they have limited ability to reject disturbances as evidenced by the continuous buildup of error over time.

The Enhanced FLC, however, has demonstrated marked improvement in transient performance as well as steady-state performance for all of the tuning techniques. The GTO optimizer applied to the Enhanced FLC as well as the MPA have almost exhibited the same level of performance as they achieved the lowest overall steady-state error. Although the Enhanced FLC tuned with the PSO did not match the level of accuracy of the GTO and MPA, it managed to significantly reduce the steady-state error compared to all of the Initial controller setups.

These findings indicate that the Enhanced FLC configuration provides a significant improvement in system stability and dynamic performance. Furthermore, both GTO and MPA optimization algorithms effectively leverage the Enhanced controller design, as they provide faster error convergence and improved system stability.

The dynamic responses of Fig. 18 clearly illustrate that the Enhanced FLC controllers tuned by means of PSO, GTO, or MPA optimizers show significant improvement over their Initial counterparts. As for frequency deviations and tie-line power, the Enhanced controllers show improved damping characteristics and a drastic reduction of steady-state error, thereby showing a significant improvement in system stability. It is also noted that the Enhanced FLC-MPA and FLC-GTO controllers show improved performance over the other controllers with better damping characteristics and zero steady-state error. Although the Enhanced FLC-PSO controllers show a significant improvement over the Initial controllers, a slightly higher Initial transience is also noted. Regarding the voltage output, it also proves the improvement achieved by the Enhanced controllers, where the voltage output shows a rapid recovery from a disturbance with minimal voltage dip.

The optimized parameter values identified for the 4th case study are summarized in Tables 18 and 19, while the corresponding time-domain dynamic performance indicators of the power system are reported in Table 20.

**Table 18:** Optimized tuning parameters of the LFC controllers for both interconnected areas.

Optimizer	Area	Initial FLC								Enhanced FLC							
		K_P_	K_I_	K_D1_	K_D2_	N_1_	N_2_	K_E_	K_CE_	K_P_	K_I_	K_D1_	K_D2_	N_1_	N_2_	K_E_	K_CE_
PSO	Area 1	100	2.00	2.00	10.0	2.00	58.7	2.00	0.1	22.92	2.00	0.93	5.39	1.82	50.33	1.77	0.05
	Area 2	83.9	2.00	2.00	6.94	2.00	96.5	1.87	0.10	40.05	1.69	1.79	5.43	1.67	74.49	1.63	0.03
GTO	Area 1	100	2.00	0.10	10.0	2.00	100	2.00	0.01	75.91	1.78	1.22	4.73	1.64	28.36	1.67	0.08
	Area 2	100	2.00	2.00	10.0	2.00	0.10	2.00	0.10	32.11	1.69	1.76	5.70	1.47	33.08	1.29	0.04
MPA	Area 1	99.8	2.00	1.99	9.59	1.99	60.6	2.00	0.02	76.40	1.98	0.13	8.89	1.86	99.76	1.85	0.08
	Area 2	48.6	2.00	1.91	3.62	0.13	100	2.00	0.01	31.07	1.61	0.77	3.22	0.10	21.68	1.98	0.10

**Table 19:** Optimized tuning parameters of the AVR controllers for both interconnected areas.

Optimizer	Area	Initial FLC								Enhanced FLC							
		K_P_	K_I_	K_D1_	K_D2_	N_1_	N_2_	K_E_	K_CE_	K_P_	K_I_	K_D1_	K_D2_	N_1_	N_2_	K_E_	K_CE_
PSO	Area 1	1.85	1.76	2.00	1.09	2.00	100.0	–	–	0.48	1.98	1.84	0.87	1.41	65.47	–	–
	Area 2	1.02	2.00	2.00	2.00	2.00	100.0	–	–	1.86	0.31	1.13	2.00	0.90	100.0	–	–
GTO	Area 1	2.00	2.00	1.30	1.72	1.29	100.0	–	–	0.44	0.28	0.38	0.37	0.35	100.0	–	–
	Area 2	0.10	2.00	2.00	2.00	2.00	100.0	–	–	0.49	0.88	1.10	0.72	1.81	34.02	–	–
MPA	Area 1	1.86	1.18	2.00	0.43	0.11	0.20	–	–	0.22	1.68	2.00	0.33	2.00	29.70	–	–
	Area 2	0.66	1.89	0.17	0.92	0.73	21.4	–	–	0.10	1.99	0.97	1.67	1.99	71.95	–	–

**Table 20:** Dynamic responses of the system.

Controller	Optimization Method	∆f_1_			∆f_2_			∆P_tie_			V_out1_				V_out2_			
		MO_∆f1_ (Hz)	MU_∆f1_ (Hz)	ESS_∆f1_ (p.u)	MO_∆f2_ (Hz)	MU_∆f2_ (Hz)	ESS_∆f2_ (p.u)	MO_∆P_ (p.u)	MU_∆P_ (p.u)	ESS_∆P_ (p.u)	MP_-V1_ (p.u)	T_r-V1 (s)_	T_s-V1 (s)_	ESS_V1_ (p.u)	MP_-V2_ (p.u)	T_r-V2 (s)_	T_s-V2 (s)_	ESS_V2_ (p.u)
Initial FLC	PSO	0.002	− 0.015	0.0009	0.001	− 0.016	0.0009	0.0004	− 0.0000	0.0003	0.42	0.11	3.0	0.00006	0.1830	0.17	3.0	0.00011
Initial FLC	GTO	0.028	− 0.046	0.0005	0.008	− 0.034	0.0005	0.0004	− 0.0005	0.0001	0.38	0.11	4.8	0.00003	0.1635	0.14	3.3	0.00006
Initial FLC	MPA	0.002	− 0.015	0.0005	0.011	− 0.036	0.0005	0.0019	− 0.0001	0.0001	0.47	0.25	4.1	0.00005	0.2201	0.20	2.6	0.00006
Enhanced FLC	PSO	0.004	− 0.106	0.0000	0.001	− 0.039	0.0000	0.0003	− 0.0034	0.0001	0.21	0.14	5.8	0.00000	0.1961	0.24	2.4	0.00001
Enhanced FLC	GTO	0.001	− 0.014	0.0000	0.002	− 0.041	0.0000	0.0027	− 0.0004	0.0000	0.00	1.32	3.7	0.00000	0.2326	0.21	3.0	0.00000
Enhanced FLC	MPA	0.001	− 0.216	0.0000	0.291	− 0.623	0.0000	0.0908	− 0.0004	0.0001	0.21	0.20	5.1	0.00000	0.1988	0.15	2.3	0.00001

Dynamic performance indices for the Initial and Enhanced FLCs are summarized in Table 20. Generally, Enhanced FLC demonstrates superior performance particularly in terms of steady-state accuracy across all parameters measured.

As for frequency deviation (Δf_1_ and Δf_2_): the Enhanced FLC exhibits no steady-state error with all optimizers, whereas the Initial FLC maintained a relatively small steady-state frequency error around 0.0005 p.u. The MPA-tuned Enhanced FLC displays the best transient behavior among the three optimization methodologies.

With respect to tie-line power deviation (ΔP_tie_), the results again indicate that the Enhanced FLC architecture achieves better performance than the Initial FLC. This improvement is very clear especially when the Enhanced FLC controller is optimized using both GTO and MPA algorithms, which are able to achieve zero steady-state error.

Furthermore, the voltage response characteristics (Vout_1_ and Vout_2_) also exhibit substantial improvements with the Enhanced FLC compared to the Initial FLC controller designs. All Enhanced FLCs, tuned using PSO, GTO, and MPA, exhibited zero steady-state voltage error. In contrast, the Initial FLC controllers provided a relatively higher steady-state voltage error compared to the Enhanced controller. In conclusion, the results from this case study indicate that the Enhanced FLC structure provides better system regulation by increasing the accuracy in the system, thereby reducing the transients in the system. In addition, the use of MPA optimizer was especially successful in taking advantage of the capabilities of the Enhanced FLC, providing robust, accurate and dynamically stable control for use in multi-area power system applications.

The optimized Integral Time Absolute Error (ITAE) and the corresponding average simulation runtime per control cycle for both the Initial and Enhanced FLC controllers under PSO, GTO, and MPA optimization algorithms are illustrated in Table 21.

**Table 21:** Comparative ITAE and simulation runtime for the initial and enhanced FLCs.

Controller	Best ITAE value			Simulation runtime (minutes/optimization run)		
	PSO	GTO	MPA	PSO	GTO	MPA
Initial FLC	62.2	54.8	54.8	20	23	29
Enhanced FLC	22.4	17.6	16.9	3	4	6

As shown in Table 21, it is obvious that the Enhanced FLC has better performance in accuracy and computational efficiency compared to the Initial FLC. It is also observed that the best ITAE value obtained by using the Initial FLC is greater than 50. On the other hand, the Enhanced FLC under all optimization techniques much lower ITAE values. For instance, when the Enhanced FLC optimized by using the MPA method, it achieves the best ITAE value all the three optimizers, reaching approximately 17. Although the PSO method obtains a slightly higher ITAE value of around 22, it still outperforms the Initial FLC under all optimization techniques.

In terms of computing efficiency, it is found that the Enhanced FLC completes a single control cycle in 3 to 6 min, whereas the Initial FLC takes 20 to 29 min.

In conclusion, the Enhanced FLC successfully achieves an effective balance compromise between precise control and lower computing requirements, particularly when optimized using the MPA or GTO approaches.

#### Results summary

Despite the random load perturbation applied to Area-1 in this case study, the Initial FLC controller is able to maintain the basic system stability. However, it is observed that all the Initial FLC controllers tuned using the three optimizers converge to a relatively higher value of ITAE. On the other hand, it is also observed that the overall system performance is significantly improved by the Enhanced FLC controller, as it is able to eliminate error completely and achieve faster convergence. In this case, both MPA and GTO used to tune the Enhanced FLC structure provide better performance than PSO. Regarding computational efficiency, it is observed that the simulation runtime is reduced by more than 80% using the Enhanced FLC compared to the Initial FLC.

From the results obtained in this case study, it can be concluded that the Enhanced FLC is more robust and resilient than the Initial FLC in controlling multi-area power systems, even under random loads.

## Statistical framework for performance assessment

### Simulation runtime and computational efficiency

The times reported in Table 22 represent the simulation runtime per optimization run, i.e., the total wall-clock time required for the metaheuristic algorithm (PSO, GTO, or MPA) to complete its search from Initial population to final iteration, as executed in MATLAB/Simulink. All simulations were performed on a system with an Intel Core i5-4300U processor (1.90–2.50 GHz) and 12 GB RAM. The runtime includes all fitness evaluations (each evaluation runs a Simulink simulation of the power system). In order to ensure objective performance benchmarking of the performance, the Initial FLC and the Enhanced FLC were subjected to the same conditions in the simulation environment.

**Table 22:** Empirical simulation runtime (minutes per optimization run).

Case Study No	Controller	Mean processing time required for a single control cycle (minutes)			Average simulation time required for a single control cycle across the three optimization algorithms (Minutes)	Reduction in simulation runtime per control cycle (modified vs initial FLC) (%)
		PSO	GTO	MPA		
1st case study	Initial FLC	16	19	24	20	85
	Enhanced FLC	2	3	4	3	
2nd case study	Initial FLC	16	19	25	20	78
	Enhanced FLC	3	4	6	4	
3rd case study	Initial FLC	17	22	28	22	84
	Enhanced FLC	3	3	5	4	
4th case study	Initial FLC	20	23	29	24	82
	Enhanced FLC	3	4	6	4	
Average						82

As shown in Table 22, the computational performance of the Enhanced FLC is significantly faster than the Initial one, as it reduces simulation time from 16–20 min taken by the Initial FLC for a single control cycle to approximately 2–4 min. This reduction corresponds to a decrease over 80% in average compared with the Initial FLC. However, the reduction in simulation runtime does not imply that the online controller executes faster, as the online execution time is determined by the fuzzy inference logic and is effectively identical for both controllers. Accordingly, the practical advantage of shorter simulation-based tuning is that the controller can be re-optimized more quickly when system parameters change, which is relevant for adaptive or self-tuning implementations.

In addition, this significant drop in simulation runtime shows that the Enhanced FLC is a much better fit for real‑time applications under the tested offline tuning budgets. Note that the per‑cycle simulation runtime does not depend on the offline optimization budget; therefore, this conclusion holds regardless of whether the controller gains were tuned with 20 or more agents.

As illustrated in Table 22, the manuscript reports empirical simulation runtime (wall-clock time) for each optimization run. However, a formal analysis of the theoretical computational complexity of the three optimizers (PSO, GTO, MPA) is not provided.

For completeness, the following brief discussion is offered. All three algorithms have a per-iteration computational complexity that scales proportionally with the product of the population size and the number of decision variables, where the population size is 20 and the number of decision variables is 16 for LFC loops and 12 for AVR loops in this study. This is because updating each agent’s position requires a number of operations that increases with the problem dimensionality. The differences lie in:

- *PSO* velocity update (two multiplications per dimension) and position update; minimal overhead.
- *GTO* requires evaluation of three distinct movement strategies (following, competition, silverback) with additional random number generations, leading to a slightly larger constant factor.
- *MPA* involves Lévy flight generation and Brownian motion calculations, which are more expensive per iteration than PSO or GTO (requires random draws from a Lévy distribution).

Despite this, the actual empirical simulation runtime illustrated in Table 22 shows that the Enhanced FLC reduces total runtime considerably because it converges in fewer iterations (often less than the maximum 30) due to a smoother fitness landscape (after fixing the crisp range shift). The ranking of optimizers by convergence speed (MPA ≈ GTO < PSO) observed in the results is consistent with their ability to escape local optima, not with per-iteration cost.

A rigorous complexity analysis (e.g., number of function evaluations to reach a given tolerance, scalability with population size, variance across multiple runs) is beyond the scope of this paper but is identified as a future research direction (Table 31).

### Sensitivity analysis of optimizer settings

In the main study, all optimizations were performed with a fixed population size of 20 agents and a maximum of 30 iterations, inherited from^71^ to ensure a fair comparison between the Initial and Enhanced FLCs. To assess whether the main conclusions (the superiority of the Enhanced FLC over the Initial FLC and the relative ranking of the three optimizers) are sensitive to these settings, an additional sensitivity analysis was conducted. This analysis was performed on Case Study 1 using the Enhanced FLC with all three optimizers. The tested configurations are summarized in Table 23.

**Table 23:** Sensitivity analysis of the ITAE of the Enhanced FLC in Case Study No.1.

Population	Max iterations	Description	Best ITAE of the Enhanced FLC using			Ranking	Best optimizer	Percentage of ITAE Reduction vs. Initial FLC (53.2) (%)	Comment
			MPA	GTO	PSO				
20	30	Original setting	11.1	11.5	20.1	MPA ≈ GTO > PSO	MPA	79.1	Stable
10	30	Half population	13.8	14.2	22.5	MPA ≈ GTO > PSO	MPA	74.1	Still valid
40	30	Double population	10.6	10.7	19.7	MPA ≈ GTO > PSO	MPA	80.1	Improved but same order
20	15	Half iterations	14.2	15.9	23.8	MPA ≈ GTO > PSO	MPA	73.3	Insufficient budget
20	60	Double iterations	10.4	10.4	18.3	MPA ≈ GTO > PSO	MPA and GTO	80.5	Stable, slightly better

As illustrated in Table 23, the following key observations can be made:

- Under all tested configurations (population size from 10 to 40, iterations from 15 to 60), the Enhanced FLC achieves a substantial reduction in ITAE compared to the Initial FLC baseline of 53.2. The observed reduction ranges from 73.3% to 80.5%, confirming that the performance advantage is not an artifact of the chosen computational budget (20 agents, 30 iterations).
- The relative ranking (MPA and GTO outperforming PSO) remains stable for all tested configurations, even when the budget is reduced (e.g., population = 10 or iterations = 15). This indicates that the original settings (population = 20, maximum iterations = 30) represent an adequate budget rather than an overly optimistic choice.

These results collectively confirm the robustness of the main conclusions across a range of optimizer settings.

### Statistical validation over multiple independent runs

Since all optimizers used in this study (PSO, GTO, MPA) are stochastic, reporting only the best ITAE value per optimizer–case study combination does not fully capture robustness or repeatability. To address this, five independent optimization runs were performed for case study No. 1 using the MPA algorithm, as shown in Table 24. Each run used the same settings (population size = 20, maximum iterations = 30) and the same simulation environment.

**Table 24:** Statistical results over 5 runs (Case Study 1, MPA optimizer).

Controller	Run number					Mean (μ)	Standard Deviation (σ)	Uncertainty (σ/√n)	Coefficient of variation (CV = σ/μ × 100%)
	1	2	3	4	5				
Initial FLC	53.2	58.5	54.1	56.3	53.9	55.20	2.179	0.974	3.95%
Enhanced FLC	11.1	12.6	12.2	13.1	11.7	12.14	0.777	0.347	6.40%
Improvement	79.14	78.46	77.45	76.73	78.29	78.01	0.937	–	–

As shown in Table 24, the Enhanced FLC consistently outperformed the Initial FLC across all five runs, with an average ITAE improvement of 78.0%. The standard deviation of the improvement is only 0.937, and the coefficient of variation (CV) of the improvement is 1.20%, indicating very high consistency and repeatability.

For the Initial FLC, the CV is 3.95%, reflecting low run‑to‑run variability. The Enhanced FLC shows a higher CV (6.40%), which is expected because its mean ITAE is much smaller. However, its absolute standard deviation (0.777) is low, demonstrating reliable performance.

This statistical validation confirms that the percentage ITAE improvement reported in this study is reliable and reproducible.

### Overall ITAE improvement across all case studies

The ITAE index for both controllers across the four case studies is summarized in Table 25.

**Table 25:** ITAE values and percentage improvement of Enhanced FLC across four case studies and three optimization methods.

Case study No	Controller	ITAE values (PSO)	ITAE values (GTO)	ITAE values (MPA)	Average across optimization methods
1st case study	Initial FLC	53.6	53.9	53.2	53.6
	Enhanced FLC	20.1	11.5	11.1	14.2
	% Improvement of Enhanced FLC	63%	79%	79%	73%
2nd case study	Initial FLC	66.1	52.2	54.8	57.7
	Enhanced FLC	25.7	17.7	14.7	19.4
	% Improvement of Enhanced FLC	61%	66%	73%	66%
3rd case study	Initial FLC	56.2	52.8	53.5	54.2
	Enhanced FLC	20	16.2	17.2	17.8
	% Improvement of Enhanced FLC	64%	69%	68%	67%
4th case study	Initial FLC	62.2	54.8	54.8	57.3
	enhanced FLC	22.4	17.6	16.9	19.0
	% Improvement of enhanced FLC	64%	68%	69%	67%
Mean of ITAE improvement (μ)		63%	71%	72%	69%
Standard deviation of ITAE improvement (σ)		1%	5%	4%	4%
Variance of ITAE improvement (σ^2^)		1.5	25.2	18.7	15.2
Relative uncertainty of ITAE improvement (σ/μ × 100%)		1.9%	7.1%	6.0%	5%

The notable observation is that the Enhanced FLC can always reduce ITAE compared with the Initial one, irrespective of the optimization strategy employed, which corresponds to an average improvement of over 70%

The values of standard deviation which range (1–5%), and relative uncertainties which range (1.9–7.1%), indicate high consistency and reliability of the results. Moreover, such figures are considered acceptable, as they are consistent with the stochastic nature of optimization algorithms without hiding significant outcomes^78,79^.

Overall and based on the results provided in Table 25, it is evident that the Enhanced FLC outperforms the Initial FLC, especially when stability, accuracy and robustness matter most.

### Percentage changes in dynamic response parameters

Table 26 includes a comparison of the percentage improvements in dynamic response parameters obtained using the Enhanced FLC controller over the Initial FLC controller, grouped by each one of the three applied optimization techniques. The median values across the four case studies are also reported to provide a robust central tendency, as medians are less sensitive to outliers than means.

**Table 26:** Percentage change in dynamic response parameters of the enhanced and initial FLC.

Case study No	Optimization Method	∆f_1_	∆f_2_	∆P_tie_	V_out1_			V_out2_		
		ESS_∆f1_ (p.u) (%)	ESS_∆f2_ (p.u) (%)	ESS_∆P_ (p.u) (%)	T_r-V1 (Sec.)_ (%)	T_s-V1 (Sec.)_ (%)	ESS_V1_ (p.u) (%)	T_r-V2 (Sec.)_ (%)	T_s-V2 (Sec.)_ (%)	ESS_V2_ (p.u) (%)
1st case study	PSO	100	100	0	− 14	− 9	100	0	− 121	100%
2nd case study	PSO	83	83	100	− 71	68	83	53	23	75%
3rd case study	PSO	80	80	0	− 75	− 4	100	− 47	− 33	75%
4th case study	PSO	100	100	67	− 27	− 93	100	− 41	20	91%
1st case study	GTO	100	100	100	− 100	− 108	100	45	− 145	100%
2nd case study	GTO	100	100	0	41	− 261	100	53	− 274	100%
3rd case study	GTO	100	100	100	− 200	− 122	100	7	0	100%
4th case study	GTO	100	100	100	− 1100	23	100	− 50	9	100%
1st case study	MPA	100	100	100	− 86	− 81	100	24	36	100%
2nd case study	MPA	100	100	100	− 211	− 3	100	− 278	− 5	100%
3rd case study	MPA	100	100	100	20	− 32	100	22	− 68	100%
4th case study	MPA	100	100	0	20	− 24	100	25	12	83%
Median of PSO		92	92	34	− 49	− 7	100	− 24	− 7	91
Median of GTO		100	100	100	− 150	− 115	100	26	− 68	100
Median of MPA		100	100	100	− 33	− 28	100	23	− 3	100
Average		97	97	78	− 77	− 50	100	8	− 26	97

Negative values indicate a deterioration in performance compared to the Initial FLC.

Since the Integral Time Absolute Error (ITAE) criterion is well suited for penalizing persistent errors, thereby encouraging zero steady-state offset and good damping, the Enhanced FLC consistently eliminates or drastically reduces steady-state errors across all case studies and optimizers, as shown in Table 26. On average, frequency deviations (Δf_1_ and Δf_2_) are reduced by 97% in steady-state error, tie-line power deviation (ΔP_tie_) is reduced by 78%, and terminal voltage (Vout_1_ and Vout_2_) is reduced by 97–100%. These results confirm that the Enhanced FLC achieves near-zero steady-state offset, which is a primary control objective for coordinated LFC-AVR, regardless of the optimizer used.

However, a single-objective ITAE formulation does not explicitly penalize peak overshoot, settling time, rise time, or control effort. Consequently, the optimization prioritizes steady-state accuracy over transient speed—a trade-off clearly visible in Table 26. While steady-state errors are virtually eliminated (positive improvements of 80–100%), some transient metrics deteriorate. For instance, voltage rise time shows negative changes (e.g., − 86% under MPA in Case 1, − 211% in Case 2), and voltage settling times occasionally increase (e.g., − 108% under GTO in Case 1). In contrast, the Initial FLC often provides shorter rise and settling times but leaves noticeable steady-state offsets. This trade-off is acceptable because the primary goal of this study is to eliminate long-term deviations caused by renewable intermittency, nonlinearities (GRC, GDB), and random load disturbances. In such operating environments, steady-state accuracy and damping of low-frequency oscillations are far more critical than the exact value of initial overshoot or rise time. Moreover, the simulation model already includes hard actuator limits (GRC, GDB) and the search space bounds (Table 5) prevent unrealistic control actions, so control effort remains within practical ranges.

Accordingly, a single-objective ITAE formulation cannot capture all desirable control attributes simultaneously. To overcome this limitation, future work should investigate multi-objective formulations that combine ITAE with additional terms such as Integral Squared Error (ISE) to penalize control energy, weighted overshoot and settling time penalties to explicitly constrain transient performance, and Pareto-based optimization to explore trade-off frontiers between steady-state accuracy, transient speed, and control effort. This direction is already listed in Table 29 (Item 9), taking into consideration that different performance indices (e.g., ITSE, IAE, or custom weighted sums) would produce different trade-offs.

Despite the inherent trade-offs of the single-objective ITAE, the Enhanced FLC is superior to the Initial FLC in all aspects of steady-state accuracy and long-term error minimization—the most critical metrics for LFC-AVR under renewable and nonlinear disturbances. The simulation time span of 300 s is sufficient to capture all relevant dynamics, so the ITAE values reliably reflect steady-state performance.

Finally, the relative ranking (MPA ≈ GTO > PSO), which is observed consistently across case studies, can be explained by the different search mechanisms of the three algorithms. The FPIDD^2^ controller optimization problem involves a high-dimensional, non-convex search space with multiple local optima, caused by nonlinear interactions among the gains (K_p_, K_i_, Kd_1_, Kd_2_, N_1_, N_2_, K_e_, KCE) and the nonlinear plant constraints (GRC, GDB). Regarding PSO, it relies on velocity clamping and global/local best attraction. In this problem, PSO frequently suffers from premature convergence: the swarm collapses to a suboptimal region before sufficiently exploring the search space, especially when the initial population is limited (20 agents). On the other hand, GTO and MPA maintain better population diversity throughout the iterations. GTO uses a combination of exploration (gorilla following) and exploitation (silverback competition) that prevents early stagnation. MPA mimics Lévy flight and Brownian motion, allowing it to escape local optima even with modest population sizes. Additionally, both GTO and MPA include stochastic jumps that are not present in standard PSO, making them more robust to the noisy fitness landscape caused by random load perturbations and renewable intermittency. This explanation is consistent with recent comparative studies on metaheuristics for power system control^78,79^.

### Statistical analysis of optimized tuning parameters (LFC and AVR loops)

Tables 27, 28, 29, and 30 present a detailed statistical analysis of the best tuning parameters for both Initial and Enhanced fuzzy logic controllers (FLC) applied to the Load Frequency Control (LFC) and Automatic Voltage Regulation (AVR) loops.

**Table 27:** Optimum tuning parameters statistics of the Initial controllers deployed in LFC loop.

Item	Initial bound		Mean	Std Dev	Min	Max	Percentile		Q_1_	Q_3_	IQR	Comments and justification
	Low	Upp					5th	95th				
K_P_	0.10	100	88.5	19.5	44.0	100	44.5	100	91.8	100	8.17	Strong convergence toward upper bound, as the middle 50% of values (25–75%) lie between (91.8 and 100)
K_I_	0.10	2.0	2	0	2	2	2	2	2	2	0	Fixed parameter, as all values are exactly 2.0 with zero variation
K_D1_	0.10	2.0	1.73	0.54	0.1	2	0.13	2	1.67	2	0.33	Values tightly clustered near upper bound, as the middle 50% of values (25–75%) lie between (1.67 and 2)
K_D2_	0.10	10.0	7.73	3.04	1	10	1.33	10	6.62	10	3.38	Bimodal distribution, as the middle 50% of values (25–75%) lie between (6.62 and 10)
N_1_	0.10	2.0	1.74	0.52	0.13	2	0.19	2	1.71	2	0.29	Values concentrated near upper limit, as the middle 50% of values (25–75%) lie between (1.71 and 2)
N_2_	0.10	100	75.3	34.2	0.1	100	0.1	100	60.1	100	39.9	Highest variability, as the middle 50% of values (25–75%) lie between (60.1 and 100)
K_E_	0.01	2.0	1.94	0.19	1.13	2	1.17	2	2	2	0	Strong central tendency toward 2.0, as most values are exactly 2.0
K_CE_	0.01	0.10	0.07	0.03	0.01	0.1	0.01	0.1	0.06	0.1	0.04	Well-distributed across the full range, as the middle 50% of values (25–75%) lie between (0.06 and 0.1)

**Table 28:** Optimum tuning parameters statistics of the Enhanced controllers deployed in LFC loop.

Item	Initial bound		Mean	Std Dev	Min	Max	Percentile		Q_1_	Q_3_	IQR	Comments and justification
	Low	Upp					5th	95th				
K_P_	0.10	100	52.4	26.7	22.9	100	23.2	94.6	31.5	76.6	45.1	Wide variability, as the middle 50% of values (25–75%) lie between (31.5 and 76.6)
K_I_	0.10	2.0	1.8	0.14	1.61	2	1.61	2	1.69	1.9	0.21	Concentrated near upper bound, as the middle 50% of values (25–75%) lie between (1.69 and 1.9)
K_D1_	0.10	2.0	1.19	0.62	0.13	2	0.21	2	0.77	1.79	1.02	Wide spread across the range, as the middle 50% of values (25–75%) lie between (0.77 and 1.79)
K_D2_	0.10	10.0	5.13	3.05	0.1	10	0.49	9.82	3.29	7.31	4.02	Moderate-to-wide distribution, as the middle 50% of values (25–75%) lie between (3.29 and 7.31)
N_1_	0.10	2.0	1.32	0.64	0.1	2	0.16	1.98	0.87	1.78	0.91	Significant variability, as the middle 50% of values (25–75%) lie between (0.87 and 1.78)
N_2_	0.10	100	69.2	27.9	21.7	100	24.6	99.9	48.9	97.1	48.22	High variability, as the middle 50% of values (25–75%) lie between (48.9 and 97.1)
K_E_	0.01	2.0	1.78	0.25	1.12	2	1.2	2	1.67	1.99	0.32	Concentrated toward upper bound, as the middle 50% of values (25–75%) lie between (1.67 and 1.99)
K_CE_	0.01	0.10	0.06	0.04	0.01	0.1	0.01	0.1	0.03	0.09	0.06	Broad distribution across the range, as the middle 50% of values (25–75%) lie between (0.03 and 0.09)

**Table 29:** Optimum tuning parameters statistics of the Initial controllers deployed in AVR loop.

Item	Initial bound		Mean	Std Dev	Min	Max	Percentile		Q_1_	Q_3_	IQR	Comments and justification
	Low	Upp					5th	95th				
K_P_	0.10	2.0	1.48	0.64	0.1	2	0.1	2	1	1.99	0.99	Moderate-to-wide variability, as the middle 50% of values (25–75%) lie between (1 and 1.99)
K_I_	0.10	2.0	1.75	0.48	0.13	2	0.17	2	1.84	2	0.16	Values concentrated near upper bound, as the middle 50% of values (25–75%) lie between (1.84 and 2)
K_D1_	0.10	2.0	1.63	0.52	0.17	2	0.22	2	1.35	2	0.65	Tendency to upper bound, as the middle 50% of values (25–75%) lie between (1.35 and 2),
K_D2_	0.10	2.0	1.32	0.6	0.21	2	0.25	2	0.95	2	1.05	Wide distribution, as the middle 50% of values (25–75%) lie between (0.95 and 2)
N_1_	0.10	2.0	1.61	0.53	0.11	2	0.12	2	1.38	2	0.62	Tendency to upper bound, as the middle 50% of values (25–75%) lie between (1.38 and 2)
N_2_	0.10	100.0	69.5	38.9	0.1	100	0.1	100	40.2	100	59.8	High variability, as the middle 50% of values (25–75%) lie between (40.2 and 100)
K_E_	–	–	–	–	–	–	–	–	–	–	–	Not used
K_CE_	–	–	–	–	–	–	–	–	–	–	–	Not used

**Table 30:** Optimum tuning parameters statistics of the Enhanced controllers deployed in AVR loop.

Item	Initial Bound		Mean	Std Dev	Min	Max	Percentile		Q_1_	Q_3_	IQR	Comments and justification
	Low	Upp					5th	95th				
K_P_	0.10	2.0	1.08	0.65	0.10	2.00	0.22	1.99	0.48	1.86	1.38	Wide variability, as the middle 50% of values (25–75%) lie between (0.48 and 1.86)
K_I_	0.10	2.0	1.42	0.6	0.28	2.00	0.29	2	1.02	1.99	0.97	Moderate-to-wide variability, as the middle 50% of values (25–75%) lie between (1.02 and 1.99)
K_D1_	0.10	2.0	1.24	0.61	0.10	2.00	0.13	2	0.77	1.84	1.07	Wide distribution, as the middle 50% of values (25–75%) lie between (0.77 and 1.84)
K_D2_	0.10	2.0	1.25	0.67	0.33	2.00	0.33	2	0.72	1.98	1.26	Wide variability, as the middle 50% of values (25–75%) lie between (0.72 and 1.98)
N_1_	0.10	2.0	1.26	0.68	0.12	2.00	0.12	2	0.65	1.95	1.3	Wide variability, as the middle 50% of values (25–75%) lie between (0.65 and 1.95),
N_2_	0.10	100.0	75.3	28.3	4.76	100	12.7	100	58.2	100	41.8	High variability, as the middle 50% of values (25–75%) lie between (58.2 and 100)
K_E_	–	–	–	–	–	–	–	–	–	–	–	Not used
K_CE_	–	–	–	–	–	–	–	–	–	–	–	Not used

The analysis included in these tables is based on 24 sets of data, including 4 case studies. Each case study is based on two areas and three optimizers applied to each FLC. These Tables 24, 25, 26 and 27) include the basic statistical indices such as the average, standard deviation, minimum, maximum values, 5th and 95th percentiles, and interquartile range (IQR). Such indicators are calculated to serve as a basis for assessing the tuning behavior of each control parameter.

The tables generally show that the Enhanced FLC controller is more able to adapt itself within the given bounds, without a string tendency to convergence to a certain bound. In addition, wider variability in the responses are observed for both Initial and Enhanced FLC deployed for AVR loop. However, for more efficient performance in future work, the predefined bounds could be slightly readjusted based on the statistical analysis of the results. The proposed upper and lower limits of controller’s tuning parameters are shown in Table 31.

**Table 31:** Proposed lower and upper bounds for future controller tuning parameters based on statistical analysis of Initial and Enhanced controller trials.

Loop	Parameter	Lower bound		Upper bound		Description
		Current	Proposed	Current	Proposed	
LFC	K_P_	0.1	25	100	150	Lower bound raised to avoid suboptimal low region. Upper bound increased to 150 as Initial Q3 (100) and 95th percentile (100) hit the ceiling
	K_I_	0.1	1.5	2	3	Lower bound raised to focus on high-performance zone. Upper bound increased as Enhanced 95th percentile (2) hits the ceiling
	K_D1_	0.1	0.1	2	3	Lower bound kept at 0.1 to maintain wide exploration. Upper bound increased as both Initial and Enhanced 95th percentile (2) hit the ceiling
	K_D2_	0.1	0.5	10	15	Lower bound raised to avoid extreme low end. Upper bound increased as Initial 95th percentile (10) and Enhanced 95th percentile (9.82) approaches the ceiling
	N_1_	0.1	0.2	2	3	Lower bound raised to focus on high-performance zone. Upper bound increased as both Initial and Enhanced 95th percentile (2) hit the ceiling
	N_2_	0.1	25	100	150	Lower bound raised, to focus on high-performance region. Upper bound increased as both Initial and Enhanced 95th percentile (100) hit the ceiling
	K_E_	0.01	1	2	3	Lower bound raised to avoid suboptimal low region. Upper bound increased as Enhanced 95th percentile (2) hits the ceiling
	K_CE_	0.01	0.01	0.1	0.15	Lower bound kept at 0.01 to maintain full exploration. Upper bound increased as both Initial and Enhanced 95th percentile (0.1) hit the ceiling
AVR	K_P_	0.1	0.1	2	3	Lower bound kept at 0.1 to maintain wide exploration. Upper bound increased as both Initial and Enhanced 95th percentile (2) hit the ceiling
	K_I_	0.1	1	2	3	Lower bound raised to avoid suboptimal low region. Upper bound increased as both Initial and Enhanced 95th percentile (2) hit the ceiling
	K_D1_	0.1	0.1	2	3	Lower bound kept at 0.1 to maintain wide exploration. Upper bound increased to 3 as both Initial and Enhanced 95th percentile (2) hit the ceiling
	K_D2_	0.1	0.3	2	3	Lower bound raised to avoid extreme low end while preserving diversity. Upper bound increased as both Initial and Enhanced 95th percentile (2) hit the ceiling
	N_1_	0.1	0.1	2	3	Lower bound kept at 0.1 to maintain wide exploration. Upper bound increased as both Initial and Enhanced 95th percentile (2) hit the ceiling
	N_2_	0.1	20	100	150	Lower bound raised to focus on high-performance region while allowing moderate exploration. Upper bound increased as both Initial and Enhanced 95th percentile (100) hit the ceiling
	K_E_	–	–	–	–	Not used in AVR loop
	K_CE_	–	–	–	–	Not used in AVR loop

## Conclusion

This paper presents a systematic investigation of the effect of crisp range modification on the performance of FPIDD^2^ controllers for coordinated LFC–AVR control in multi-area power systems. Unlike conventional approaches that focus on tuning controller gains or membership functions, this study identifies crisp range selection, particularly re-centering the Zero membership function, as a key design parameter while preserving the fuzzy rule base. The main contribution lies in introducing and exploiting this additional tuning dimension, which has been largely overlooked in fuzzy control literature.

To validate the proposed approach, the controllers are evaluated across four case studies using three metaheuristic optimization algorithms, including PSO, GTO, and MPA. To ensure realistic assessment, practical operating conditions are also considered, including renewable energy intermittency and electric vehicle participation.

The results demonstrate that crisp range tuning significantly enhances controller performance by reducing ITAE, steady-state error, and computational time, while improving sensitivity and damping characteristics. MPA and GTO consistently outperforms PSO in tuning FPIDD^2^ controllers for LFC–AVR systems.

The achieved improvements are substantial, including up to 69% reduction in ITAE, elimination of steady-state error, and approximately 80% reduction in simulation runtime, enabling faster retuning in adaptive control applications. Meanwhile, the online execution time remains extremely low (microsecond scale) for both controller variants, confirming their suitability for real-time implementation.

However, these gains are accompanied by acceptable trade-offs, such as increased voltage rise and settling times in some configurations, reflecting the emphasis of the ITAE objective on steady-state accuracy.

Accordingly, the proposed technique provides a simple and practical tuning approach for enhancing the performance of FPIDD^2^ controllers in two-area power system applications.

Despite promising results, several limitations remain. The study is limited to a two-area power system, which restricts scalability assessment. Therefore, validation on larger and more complex networks is necessary. Additionally, the offline tuning process for metaheuristic optimization is computationally intensive and depends on trial-and-error initialization.

Another limitation concerns the objective function. As discussed earlier, the use of a single-objective ITAE function biases optimization toward steady-state performance and damping, potentially at the expense of control effort and transient speed. To overcome this limitation, future work should investigate multi-objective formulations incorporating indices such as ISE and IAE to better balance overshoot, rise time, and steady-state error.

In addition, the shift magnitude is determined through a one-dimensional parametric sweep. For practical applications, this sweep can be conducted offline using a validated plant model; if system dynamics vary over time, an adaptive mechanism can be developed to update the crisp range online.

This study also does not include comparisons with other advanced controller types (e.g., adaptive, MPC, neural network, or type-2 fuzzy controllers). Although such comparisons are beyond the current scope, they represent an important direction for future work (see Table 32).

**Table 32:** Structured future research directions for enhancing the proposed FPIDD^2^-Based LFC–AVR Control framework.

Category	No	Research focus	Current study’s scope	Proposed direction for advancement
Controller architecture and adaptability	1	FLC granularity	Fixed 5 linguistic variables	Investigate optimal variable count (3, 7, 9) via sensitivity analysis
	2	Core tuning mechanism	Offline optimization of crisp ranges	Develop online adaptive algorithms for real-time crisp-range adjustment
	3	Self-tuning capability	Static controller parameters	Develop self-learning FPIDD^2^ controllers capable of online parameter adaptation
	4	Membership function form	Triangular MFs	Explore Gaussian, trapezoidal, and adaptive MF types
	6	Fuzzy input variable sets	Error (E) and Change of Error (CE)	Incorporate an integral-based fuzzy input (I) to enhance steady-state accuracy
	7	Type-2 fuzzy logic control	Type-1 fuzzy controller	Investigate interval Type-2 fuzzy controllers to better manage uncertainties in nonlinear power-system dynamics
Optimization and performance framework	8	Algorithm benchmarking	Tested with PSO, GTO, MPA	Expand suite (e.g., DE, HHO, SSA) for robust comparative analysis
	9	Objective formulation	Single-objective (ITAE)	Adopt multi-objective functions (e.g., ITAE + ISE + overshoot penalty, or Pareto-based optimization) to explicitly trade off steady-state error, control effort, and transient speed
	10	Constraint handling	Fixed parameter bounds	Implement adaptive boundary mechanisms
	11	Population size and iteration budget	Limited number of search agents and iterations (20 agents, 30 iterations)	Investigate the impact of larger population sizes and extended iteration (test number of search agents = 50, iterations = 100 or more for further improvement)
	12	Optimization robustness	Deterministic evaluation conditions	Evaluate optimization performance under stochastic disturbances and parameter uncertainties
	13	Computational efficiency	Offline optimization	Investigate surrogate modeling or reduced-order optimization techniques to reduce computational burden
Robustness	14	Step load penetration (SLP)	Fixed at 2.5%	Test the effect of larger load shocks (10–20%)
Validation and practical deployment	15	System scale	Two-area interconnected power system	Validate on larger, heterogeneous, and renewable-rich networks
	16	Renewable penetration level	Moderate RES integration	Investigate controller performance under ultra-high renewable penetration scenarios
	17	Real-time validation	Simulation-based validation	Hardware-in-the-loop testing using platforms like OPAL-RT or dSPACE, including communication delays and hardware nonlinearities
	18	Smart-grid integration	Conventional interconnected system model	Evaluate controller performance within smart-grid frameworks incorporating distributed energy resources and energy management systems
	19	Energy storage integration	Not considered	Investigate coordinated control with battery energy storage systems (BESS)
	20	Experimental/field data validation	Simulation-only (validated nonlinear model, no physical hardware)	Develop a scaled laboratory microgrid (e.g., two-area system with real controllers); test with measured renewable generation and load profiles; compare simulation predictions with experimental results. Alternatively, use hardware-in-the-loop (HIL) with recorded field data from a real power system
Comparison with other controllers	21	Benchmarking against other advanced controller types	Only compared Enhanced FLC vs. Initial FLC (same architecture)	Systematic comparison with adaptive controllers, MPC, sliding mode control, neural network controllers, type‑2 fuzzy, and reinforcement learning under identical test scenarios (identical disturbances, nonlinearities, and performance metrics)
Computational complexity	22	Formal complexity analysis of optimizers for LFC-AVR tuning	Empirical simulation time reported (Table 22); no theoretical complexity analysis	Conduct convergence rate analysis, scalability tests with population size/dimensionality, memory usage profiling, and relative complexity comparisons using standard metrics (e.g., number of function evaluations to specified ITAE threshold)

Furthermore, the results are based entirely on simulation. Therefore, the reported improvements should be interpreted as simulation-based evidence. Although the model includes nonlinearities such as GRC, GDB, and renewable intermittency, real-world factors such as actuator dynamics, communication delays, and measurement noise may affect performance. Consequently, experimental validation using hardware-in-the-loop platforms (e.g., OPAL-RT, dSPACE), scaled microgrids, or real system data is required to confirm real-time feasibility. This limitation is addressed in Table 32.

Finally, a detailed theoretical complexity analysis, including scalability and convergence rate comparisons, is not included but is noted as a future direction.

Addressing these limitations forms the foundation for future research toward practical deployment. A structured summary of these directions is provided in Table 32.

## Data Availability

All data generated or analyzed during this study are fully included within this article.

## List of symbols

∆f_1_: The amount of frequency deviation in the first area (Hz)
∆f_2_: The amount of frequency deviation in the second area (Hz)
∆P_tie_: The tie-line’s power deviation (p.u.)
∆V_1_: The amount of voltage deviation in the first area (p.u.)
∆V_2_: The amount of voltage deviation in the second area (p.u.)
AVR: Automatic voltage regulator
B_1_, B_2_: Frequency bias coefficients
CE: The rate of change of error
E: Error of fuzzy controller
E_ss_: Steady state error
EV: Electrical Vehicle
ESS_∆f1_: First area steady-state error of frequency deviation
ESS_∆f2_: Second area steady-state error of frequency deviation
ESS_∆P_: Steady state error of tie-line power deviation
ESS_V1_: First area steady-state error of terminal voltage
ESS_V2_: Second area steady-state error of terminal voltage
FPIDD^2^: Fuzzy-based proportional integral derivative second (double) derivative controller
GTO: Gorilla troops optimization
ITAE: Integral time absolute error
K_D1_, K_D2_: Derivative gains
K_E_, K_CE_: Fuzzy controller inputs scaling factors
K_I_: Integral gain
K_Line_: Synchronizing coefficient
K_P_: Proportional gain
LFC: Load frequency control
M_p-V1_: Voltage overshoot of the first area
M_p-V2_: Voltage overshoot of the second area
MO_∆f1_: Peak frequency deviation overshoot of the first area
MO_∆f2_: Peak frequency deviation overshoot of the second area
MO_∆P_: Peak amplitude of overshoot in tie-line power deviation
MU_∆f1_: Maximum frequency deviation undershoot of the first area
MU_∆f2_: Maximum frequency deviation undershoot of the second area
MU_∆P_: Maximum undershoot of tie-line power
MPA: Marine Predator Algorithm
N_1_, N_2_: The coefficients of the filters
PID: Proportional integral derivative controller
PIDD^2^: Proportional integral derivative double derivative controller
PSO: Particle Swarm optimization
PV: Photovoltaics
RESs: Sources of renewable energy
RLP: Random load perturbation
SLP: Step load perturbation
T_r-V1_: Terminal-voltage rise time of the first area (s)
T_r-V2_: Terminal-voltage rise time of the second area (s)
T_s-V1_: Terminal-voltage settling time of the first area (s)
T_s-V2_: Terminal-voltage settling time of the second area (s)
